# Diffusiophoresis in Polymer and Nanoparticle Gradients

**DOI:** 10.1021/acs.jpcb.4c00985

**Published:** 2024-06-05

**Authors:** Burak Akdeniz, Jeffery A. Wood, Rob G. H. Lammertink

**Affiliations:** Soft Matter, Fluidics and Interfaces, MESA+ Institute for Nanotechnology, University of Twente, P.O. Box 217, 7500 AE Enschede, The Netherlands

## Abstract

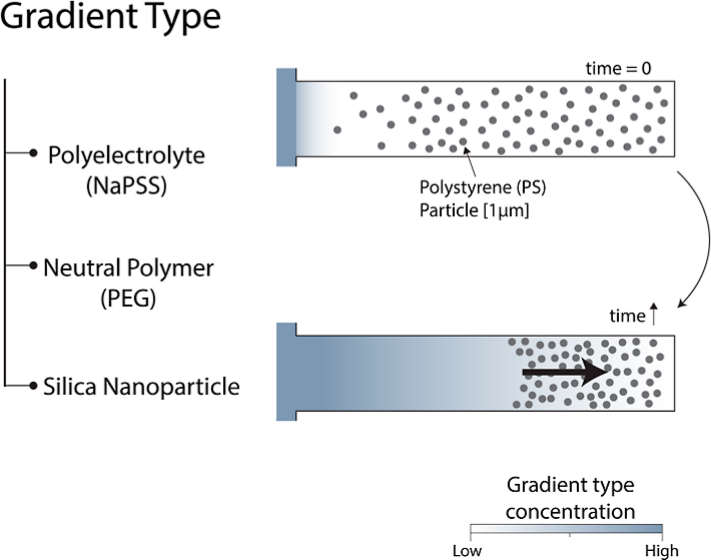

Diffusiophoresis is the movement of the colloidal particles in response to a concentration gradient and can be observed for both electrolyte (e.g., salt) and nonelectrolyte (e.g., glucose) solutes. Here, we investigated the diffusiophoretic behavior of polystyrene (PS–carboxylate surface) microparticles in nonadsorbing charged and uncharged solute gradients [sodium polystyrenesulfonate (NaPSS), polyethylene glycol (PEG), and nanoscale colloidal silica (SiO_2_)] using a dead-end channel setup. We compared the diffusiophoretic motion in these gradient types with each other and to the case of using a monovalent salt gradient. In each of the nonadsorbing gradient systems (NaPSS, PEG, and SiO_2_ nanoparticles), the PS particles migrated toward the lower solute concentration. The exclusion distance values (from the initial position) of particles were recorded within the dead-end channel, and it was found that an increase in solute concentration increases exclusion from the main channel. In the polyelectrolyte case, the motion of PS microparticles was reduced by the addition of a background salt due to reduced electrostatic interaction, whereas it remained constant when using the neutral polymer. Particle diffusiophoresis in gradients of polyelectrolytes (charged macromolecules) is quite similar to the behavior when using a PEG gradient (uncharged macromolecule) in the presence of a background electrolyte. Moreover, we observed PS microparticles under different concentrations and molecular weights of PEG gradients. By combining the simulations, we estimated the exclusion length, which was previously proposed to be the order of the polymer radius. Furthermore, the movement of PS microparticles was analyzed in the gradient of silica nanoparticles. The exclusion distance was higher in silica nanoparticle gradients compared to similar-size PEG gradients because silica nanoparticles are charged. The diffusiophoretic transport of the PS microparticles could be simulated by considering the interaction between the PS microparticles and silica nanoparticles.

## Introduction

A solute gradient can induce fluid flow along a solid surface. This feature was first explored by Derjaguin et al.^1−4^ and is called diffusio-osmosis. This phenomenon is also observed at the surface of solid particles, which leads to the movement of the solid particle, referred to as diffusiophoresis. The interaction between the solute and the surface determines the direction and magnitude of the fluid flow.^5^ The theoretical velocity description depends on the nature of the solute gradient. There are two broad divisions in solutes: nonelectrolyte^6^ and electrolyte.^7^ The driving force in both cases is the concentration gradient; however, the underlying transport mechanism differs. For example, diffusiophoretic/diffusio-osmotic velocity scales with ∇*c* or ∇*c*/*c* for nonelectrolyte and electrolyte solutes, respectively. The velocity is generally higher in electrolyte cases at low concentrations than in nonelectrolyte solutes due to the relative gradient difference that plays a role in electrolyte cases.

For nonelectrolyte solute gradients, the velocity description depends on the interaction between the solute and the solid surface,^8^ represented by the total potential energy. The interaction may be van der Waals, hydrophobic, dipole interaction, or excluded volume effects.^5,9^ Tracer particles can move toward (or escape from) the concentration-rich region mainly when the nonelectrolyte molecule is adsorbed on (or excluded from) the surface.^10,11^ Anderson^5^ provided the diffusiophoretic velocity description for the case of steric exclusion. The diffusiophoretic velocity can be written as in eq 1.
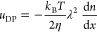
1where *k*_B_ is the Boltzmann constant, *T* is the absolute temperature, *n* is the number concentration of solute, η is the viscosity of the solution, and λ is the exclusion length. This diffusiophoretic velocity of the particle is always toward the lower concentration, while the diffusio-osmotic velocity is toward a high concentration since they are mathematically equal, but in the opposite direction when the size of the particle is larger than the solute molecule (also when the surface curvature is negligible)^12^ (*u*_DO_ = −*u*_DP_).

The exclusion length is an important parameter as the velocity scales with λ^2^. Experimentally, glucose,^13^ dextran,^14^ and PEG^10,15^ were used as nonelectrolyte solutes in diffusiophoresis and diffusio-osmosis experiments. In the case of polymers, λ is the scale of polymer radius.^12^ The radius value in diffusiophoretic experiments has previously been associated using the radius of gyration (*R*_g_),^15^ which is the average distance between the monomer unit and the center of mass of the polymer,^16^ or with the hydrodynamic radius (*R*_h_),^10,14^ which is the equivalent sphere in terms of the dynamic properties of a polymer.^16^ There are several experimental methods^16−22^ for obtaining both the hydrodynamic radius and the radius of gyration. It is also possible to determine the end-to-end distance (*R*_ee_) from coupling experiments and molecular dynamic simulations to understand the conformation of polymers.^23^ Here, we compared the fitted radii results from diffusiophoretic experiments with the available radii.

This study extends the observations of diffusiophoresis to polyelectrolytes. Polyelectrolytes are polymers with charged groups and can be found in nature, including DNA, RNA, proteins, and polysaccharides.^24^ Previously, particle movement through a polyelectrolyte gradient has been described in terms of the nonelectrolyte diffusiophoretic movement.^25^ Here, we compared the similarities and differences between polyelectrolyte gradients and monotonic salt/neutral polymer gradients.

The gradient of smaller particles facilitates the diffusiophoretic movement of larger particles in the opposite direction of small particle gradients.^26,27^ This movement is important in drying thin films and stratification.^26−28^ Several theories^26,27^ have been used to explain this observation. One suggestion is to use eq 1.^27^

In this article, we aim to study the movement of PS microparticles under a gradient of polyelectrolytes, neutral polymers, and nanoparticles. In the first part, we showed the movement of PS microparticles under polyelectrolyte gradients of various concentrations and molecular weights. We compared the observations with those in salt and neutral polymer gradients. We also analyzed the movement of the PS microparticles under background salt. We then showed the movement of the particles under a poly(ethylene) glycol gradient for different concentrations and molecular weights. We performed simulations to determine the exclusion length and relate the obtained values to the previous polymer size measurements (*R*_h_, *R*_g_, or *R*_ee_). Finally, we investigated the PS microparticle movement under nanoparticle gradients and performed simulations based on different theories to explain the experimentally observed behavior.

## Experimental Section

### Materials

Polyethylene glycol (PEG) with different molecular weights (MW ≈ 400, 2000, 3000, 4000, 6000, 35,000, and 1,000,000 Da) and poly(sodium 4-styrenesulfonate) (NaPSS, MW ≈ 70,000 and 1,000,000 Da) were purchased from Merck (The Netherlands). PEG solutions were used without purification, whereas NaPSS solutions were used with and without purification by using a dialysis bag against Milli-Q water. For dialysis, we have used two bags with a molecular-weight-cutoff of 14,000 Da (dialysis tubing cellulose membrane, D 9402-100 FT from Sigma-Aldrich, The Netherlands) and 100,000 Da (Biotech CE Tubing, from Spectrum, California, United States).

RTV-615 A (Permacol B.V., Ede, The Netherlands); prepolymer (1020 kg/m^3^) and RTV-615 B (Permacol B.V., Ede, The Netherlands); curing agent (990 kg/m^3^) were used to produce polydimethylsiloxane (PDMS). Sodium chloride (NaCl) (>99.96%) was purchased from AkzoNobel (The Netherlands). FluoSpheres polystyrene (PS)–carboxylate; 1.00 μm (2.0 wt %, abs/em = 580/605 nm) was purchased from Thermo Fischer (United States). Silica nanoparticles (sicastar, 43-00-101); 10 nm (25 mg/mL), and (micromer, 30-00-301); 30 nm (25 mg/mL) were purchased from Micromod (Rostock, Germany).

### Device Fabrication

PDMS is prepared by mixing the prepolymer (RTV-615 A) and the curing agent (RTV-615 B) in a ratio of 10:1. The prepolymer and the curing agent are blended for at least 5 min to obtain a uniform mixture. Then, the mixture is placed in a desiccator to degas for at least half an hour, and it is poured onto the two Si-wafer molds [Si-wafer without any structure (flat) and Si-wafer with positive dead-end channel structure] and degassed again to remove all bubbles. The PDMS mixture was cured for 4 h at 80 °C in an oven, after which it could be peeled from the wafers. After activating the flat and structured PDMS surfaces using O_2_ plasma, a Femto plasma cleaner (Diener Electronic GmbH, Ebhausen, Germany), for 12 s at 100 W, they were bound to each other. Prepared microfluidic devices were soaked in deionized water (Milli-Q) prior to experiments in order to reduce water permeation through the PDMS walls.^29^

**Figure 1:**
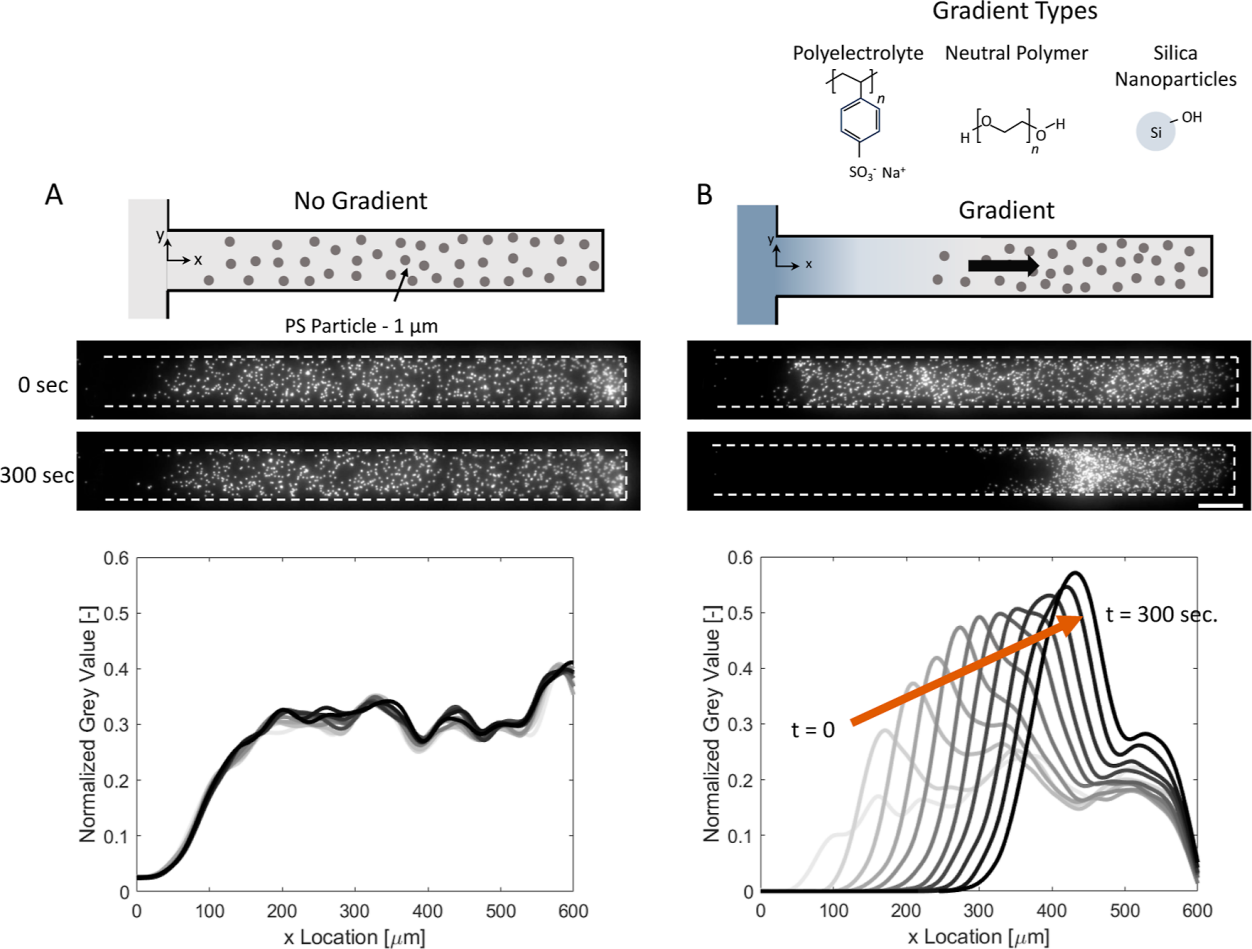
Illustration of the experiments and typical results. (A) Control experiment contains a dead-end channel, which was filled with 1 μm fluorescent PS particles in Milli-Q water. Both the main and dead-end channels are filled with Milli-Q water. (B) For the diffusiophoresis experiments, the main channel was filled with different solutes: polyelectrolyte—NaPSS, neutral polymer—PEG, or silica particles. Therefore, we obtain a transient gradient of these solutes (illustrated in blue). The chemical structures of the used solutes are given above. Microscope images of the initial situation and 300 s after gradient initiation are shown. Scale bar = 50 μm. The normalized gray values are obtained from the microscope images for *t* = 0–300 s with 30 s intervals.

The microfluidic device contains a main channel connected with dead-end channels (schematic of a dead-end channel, Figure 1). The main channel is 600 μm wide and 100 μm high. The dead-end channel is 50 μm wide (*W*), 10 μm high (*H*), and 600 μm long. The uncertainty of dimensions is around 1 μm.

### Characterization

A Zetasizer Nano-ZS (Malvern Panalytical B.V., Almelo, The Netherlands) device was used to determine the size of the silica particles and electrophoretic mobilities, which are correlated with the zeta potential [***U***_electrophoresis_ = 2εε_0_ζ *f*(κ*a*)***E***/(3η)]. Henry’s function *f*(κ*a*) is estimated by Swan’s approach for each case^30^ to account for particle size and electrolyte concentration. We determined the zeta potential of particles by measuring the electrophoretic mobility of particles. (≈0.005% w/v), and the electrophoretic mobility of particles was then measured.

UV–vis spectroscopy (Shimadzu UV-1800, Japan) was used to analyze the styrenesulfonate amount^31^ at λ_max_ = 225 nm, the maximum absorbance wavelength for NaPSS. A calibration curve was obtained with known concentrations of NaPSS, which was further used to determine the NaPSS amount in the dialysis bag.

Ion chromatography (858 Professional Sample, Processor, Eco IC, Metrohm) was used to analyze the ion types in the permeate side of the dialysis of NaPSS solutions. The setup contains an anion column (Metrosep A Supp 17 150/4.0), a suppressor for the anion analysis, and a cation column (Metrosep C 6-150/4.0). The eluent consisted of aqueous solutions of 4 mM HNO_3_ for the cation column, 5 mM Na_2_CO_3_ + 0.2 mM NaHCO_3_ for the anion column, and 0.3 M H_3_PO_4_ as suppressor solution.

A portable conductivity meter (Cond 3120, WTW GmbH) was used to measure the conductivity of the permeate side of the dialysis bag. Gel permeation chromatography (Agilent 1200 Series/1260 Infinity), which uses two columns (Supreme 8 × 300 mm, 1000–30 Å, 10 μm, Polymer Standard Service GmbH) in series and a refractive index detector (Agilent 1150 HPLC G1362A RID detector), was used to analyze the molecular weight distribution and the polydispersity index of the used PEG solutions. For that, approximately 1 g/L of PEG solutions in Milli-Q water were prepared.

A viscometer (MCR 502 with CP50-1 cone-plate geometry spindle, Anton Paar) was used to analyze the viscosity of PEG-400 solutions. The measurement was performed at 1–1000 s^–1^ shear rates (increasing and decreasing order) at 20 °C.

### Diffusiophoresis Experimental Protocol

Prior to the diffusiophoretic experiments, the PS particle suspension was cleaned at least 3 times. In the cleaning step, we added 5 mL of Milli-Q water to an aqueous particle solution, and we used a centrifugal device (Corning LSE, New York, United States) to sediment the particles (6000 rpm for 15 min). We discard the supernatant and continue the cleaning of the particles by adding Milli-Q water. This step is repeated at least 3 times before the experiment. PEG and silica particles were used without a purification step. NaPSS solutions were used in diffusiophoretic experiments without and with the cleaning step by dialysis. After cleaning the NaPSS solution, we used UV–vis spectrometry to analyze the NaPSS concentration in the dialysis bag.

In the diffusiophoretic experiments, a plastic syringe is used to fill the dead-end channel with the ∼0.1% w/w particle solutions. Then, an air bubble was passed through to clear the main channel and avoid mixing the macromolecular solution with the particle suspension beforehand. Then, the macromolecular solution (either a neutral polymer, nanoparticle, or polyelectrolyte) was filled into the main channel, and the flow was stopped after the contact between the two liquids. An inverted microscope (Zeiss Axio Observer Z1, Carl-Zeiss, Jena, Germany) was employed with a 20 × *f*/0.4 objective (distance of field is 5.8 μm, Zeiss LD Plan-Neofluar, Carl-Zeiss) to visualize the particle movement inside the dead-end channel. The particle motion was captured by a CCD camera (Hamamatsu, Japan) with a 2048 × 700 pixels resolution. The images are sequentially captured for 5, 6, 10, or 60 min in 1 or 10 frames-per-second. The set of microscope images was analyzed in ImageJ,^32^ an open-source image analysis software. Then, the software program written in Matlab was used to analyze the gray value [0 (black)–256 (white)]. The normalized gray value is calculated by dividing the gray value by 256. Using the video gathered from ImageJ, the averaged gray value is determined for each *x* position (see Figure S2). From the image, we found that the middle location between the maximum and minimum gray values was used to define the position of the edge of the particle population (exclusion distance). It is important to note that the initial value of the exclusion distance changed from experiment to experiment. Therefore, the starting position may vary slightly.

## Results and Discussion

We performed diffusiophoresis experiments in a dead-end channel (Figure 1), which is commonly used in the analysis of diffusiophoresis and diffusio-osmosis.^33−42^ The gradient through the dead-end channel can be created by replacing the solution in the main channel. In this article, the PS particle suspension in Milli-Q water (particle zeta potential ∼ −80 mV at 1 mM NaCl and varies with salt concentration^35^) was initially placed in the dead-end channel (approximately 300–500 particles, corresponding to a particle volume fraction of approximately 0.004). The gradient-forming solutes were initially located in the main channel initially (Figure 1).

The control and concentration gradient experiments are shown in Figure 1A,B. In the control experiments, no gradient is present between the main and dead-end channels (both are Milli-Q water). The normalized gray value, which is shown at the bottom of Figure 1A, indicates that the particles hardly move according to their initial position. Only the diffusion of particles drives the particles (*D*_p_ ∼ 10^–13^ m^2^/s). On the other hand, in experiments with a gradient present, the particles move into the dead-end channel.

Below, we investigate three gradient types in detail. First, we present the experiments in the presence of polyelectrolytes, which are charged macromolecules. Then, we studied the use of a neutral polymer gradient (PEG gradient) with different concentrations (same molecular weight) and different molecular weights (same molar concentration). Finally, we investigated gradients based on silica nanoparticles of approximately the same size as the PEG molecule.

### Polyelectrolyte (Polysalt) Gradients

We prepared a polyanion (NaPSS—chemical structure Figure 1), which is a strong polyelectrolyte, solution in Milli-Q water and in a 10 mM NaCl solution. The bulk salt concentration influences the shape and size of the polyelectrolytes.^43,44^ Moreover, the negatively charged polyelectrolyte is electrostatically repelled from the particle and channel wall surfaces.

**Figure 2:**
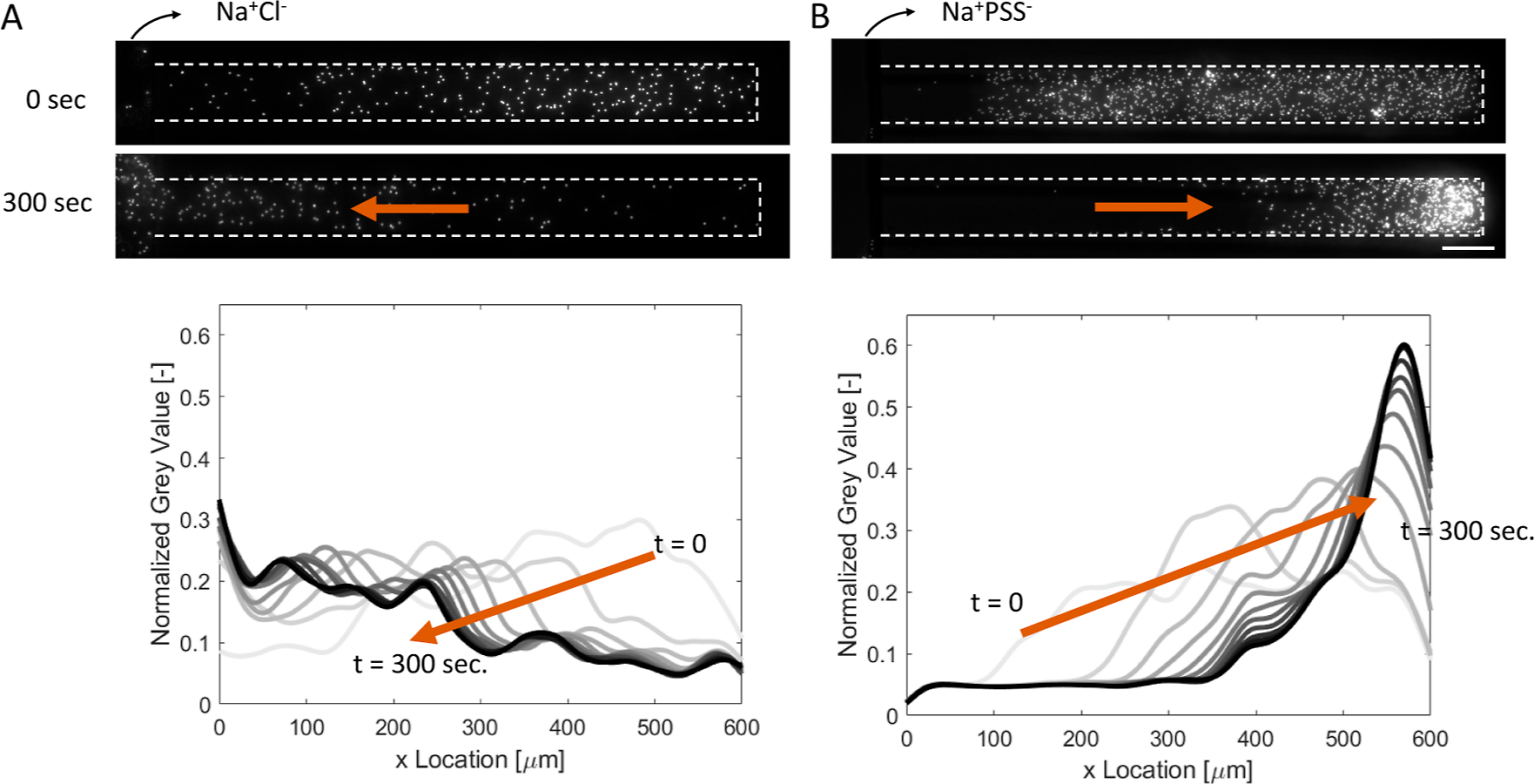
Comparison between the salt and polyelectrolyte gradients. The main channel contained (A) 10 mM NaCl and (B) 1 g/L NaPSS (1,000,000 Da) solutions. Microscope images at *t* = 0 and 300 s after contact are shown above. Particle movements are shown with the arrows. Scale bar = 50 μm. The normalized gray values are given for both cases in 0–300 s for 30 s intervals below.

Diffusiophoretic experiments were performed using gradients of salt (NaCl) and polysalt (NaPSS) (Figure 2). PS particle movement in NaPSS is opposite to that in NaCl. Figure 2A illustrates the movement of particles toward the high salt concentration. This behavior was previously observed^36^ and can be explained by two competing effects that govern the diffusiophoretic velocity:^5,7^ electrophoresis (∼βζ) and chemiphoresis (∼ln cosh ζ). The electrophoretic term is due to the diffusivity contrast between the ion pairs [β = (*D*_+_ – *D*_–_)/(*D*_+_ + *D*_–_) for symmetric electrolytes or β = (*D*_+_ – *D*_–_)/(*Z*_+_*D*_+_ – *Z*_–_*D*_–_) for asymmetric electrolytes]. That creates a spontaneous electric field to maintain electroneutrality, which drives the particle to either a higher or lower concentration depending on the sign of ζ and β.^45^ The electrophoresis term is positive (β = −0.207 for NaCl and ζ < 0) in the NaCl case. This contribution directs the particles toward higher NaCl concentrations. In addition, the chemiphoretic term is due to the nonuniform accumulation of ions around the particle, which creates an osmotic pressure difference. This component is always positive for monovalent salts and also drives the particle in the direction of higher concentration. The addition of these two components causes the particles to move toward a higher concentration of NaCl. This is simply what we also observed in the experiments.

In the case of polysalts (1 g/L dialyzed NaPSS solution), shown in Figure 2B, the particles move in the opposite direction compared to the NaCl case. The asymmetric analytical description of the diffusiophoretic velocity^46^ was used to describe this case. According to our calculation (Supporting Information S1.3), the electrophoretic term (β = 0.03 for NaPSS and ζ < 0) is negative and drives the particle to the lower concentration, while the chemiphoretic term is positive and drives the particle to the higher concentration. The chemiphoretic term is stronger than the electrophoretic term due to the small β term. Thus, particles were expected to move toward the higher concentration direction. It is important to note that the effective valency may change due to counterion condensation (increases β values). The particle direction predicted by eqs S9 and S10 in the Supporting Information depends on the degree of counterion condensation (effective valency) and the zeta potential of the particle. However, the underlying assumptions in the derivation of this analytic estimation are finite ion size, dielectric decrement, and the absence of surface conduction.^46^ The polyelectrolyte size is either similar or larger than the size of the Debye length, which does not match the first assumption.

Schulz et al.^25^ explained similar behavior for a negatively charged particle and poly(acrylic acid) by neutral polymer explanation by Sear and Warren^12^ (interfacial tension difference). These opposite directions can be attributed to the exclusion behavior of the polyelectrolyte. The polyelectrolyte (NaPSS) molecule is negatively charged, and the particle is also negatively charged. This creates a repulsive interaction between the particle and the polyelectrolyte, causing the particle to move in the direction of lower concentration, similar to eq 1.

In the experiments, the polyelectrolyte purification process is a critical step in reducing any additional effects. When the experiments were performed without the dialysis purification step, the particles in a dead-end channel showed three phases: the evacuation phase, the stationary phase, and the exclusion phase (Supporting Information Section S4, Figures S10 and S11). After contact between the polysalt solution without dialysis and the particles in Milli-Q water, the particles first move toward the main channel (similar to the NaCl case discussed earlier) for the first 60 s, referred to as the evacuation phase. Then, particles in the dead-end channel show no preferential direction during the stationary phase, which lasts between 60 and 120 s. Finally, particles are excluded from the main channel and move further into the dead-end channel (exclusion phase in 120–540 s). An increase in the polyelectrolyte concentration in the main channel magnifies both the evacuation and exclusion steps. This tendency is due to impurities present in the polyelectrolyte solution. By introducing a cleaning step (using a dialysis bag; see Experimental Section), the initial movement of particles can be eliminated, allowing the identification of the effect of polyelectrolyte gradients.

We analyzed the impurities by analyzing the dialysis permeate using an ion chromatography setup (Supporting Information S4). Figure S12 shows the variety of impurities that caused the evacuation phase. The most common identified impurities were Na^+^ and Br^–^/Cl^–^/SO_4_^2–^.

**Figure 3:**
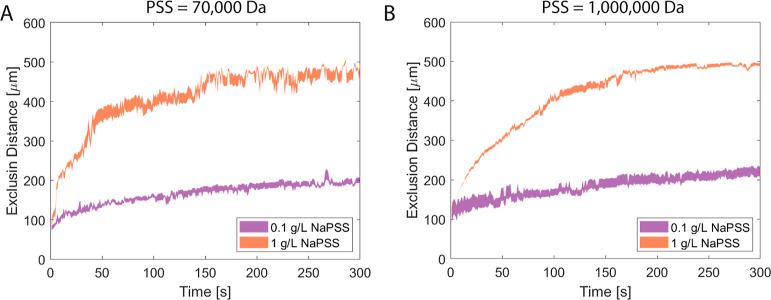
Results of diffusiophoretic experiments performed with 70,000 and 1,000,000 Da NaPSS solutions with the same mass concentrations; 0.1 and 1 g/L. (A) Excluded distance values in time for the 0.1 and 1 g/L 70,000 Da NaPSS solutions. (B) Excluded distance values in time for the 0.1 and 1 g/L 1,000,000 Da NaPSS solutions. The color-filled areas show the standard error of 3 or 4 experiments.

Figure 3 displays the exclusion distance of particles under a gradient of purified 70,000 and 1,000,000 Da NaPSS at mass concentrations of 0.1 and 1 g/L. For both molar masses, larger exclusion values were observed at higher mass concentrations of NaPSS. However, the particles started to aggregate^47^ beyond a certain concentration of NaPSS (∼>10 g/L), likely due to the depletion interaction.^48^ At low concentrations (∼<0.05 g/L), the particles did not move at all. Based on the phase diagram of NaPSS,^49^ the NaPSS solutions in our experiments are in the dilute regime.

Figure 3A shows the exclusion distance over time for a 70,000 Da molecular weight NaPSS gradient, whereas Figure 3B shows the exclusion distance for a 1,000,000 Da NaPSS gradient. The exclusion distance and the time behavior are similar when comparing the 70,000 and 1,000,000 Da NaPSS cases with the same mass concentrations. Besides a difference in the absolute polymer concentration gradient, the diffusivity, viscosity, and density of the solution change with the molecular weights. We observed some behavioral differences when comparing the PEG (see below) with different molecular weights but the same mass concentrations. (1) The mass concentration used with NaPSS is lower than with PEG to observe similar exclusion distance over time behavior (see below). (2) The behavior of the exclusion distance at 300 s for NaPSS is similar to each other for the same mass concentration, but different molecular weights (70,000 and 1,000,000 Da—Figure 3). However, we did not observe similar behavior in the PEG gradient (see Supporting Information Figure S20). For PEG, the behavior can be explained with eq 1 (λ increases and d*n*/d*x* decreases, but not on the same scale). These differences in behavior show that the underlying mechanism is different from the neutral PEG case.

**Figure 4:**
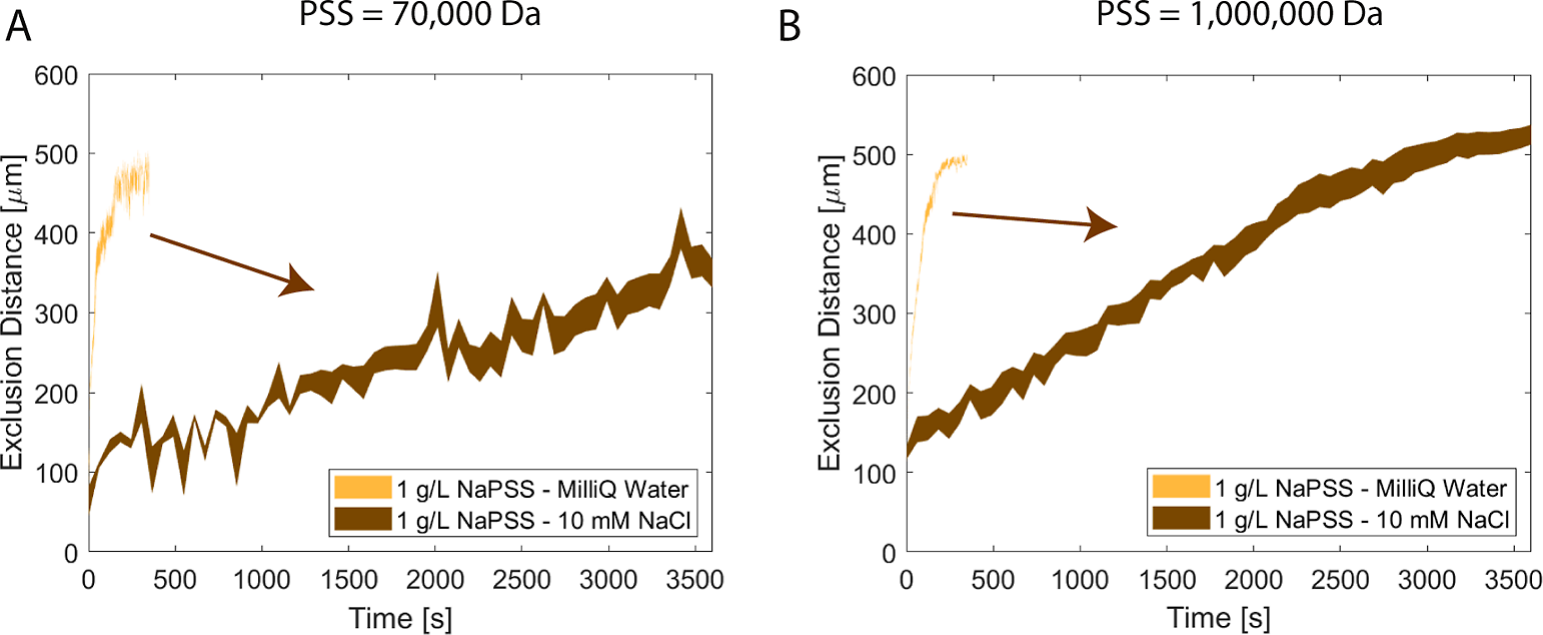
Results of diffusiophoretic experiments performed with 70,000 and 1,000,000 Da NaPSS solutions with 1 g/L and 10 mM NaCl background solute. The particle solutions were also in 10 mM NaCl. (A) Excluded distance values in time for the 1 g/L 70,000 Da NaPSS solutions with and without 10 mM NaCl background solution are given. (B) Excluded distance values in time for the 1 g/L 1,000,000 Da NaPSS solutions with and without 10 mM NaCl background solution are given. The color-filled areas show the standard error of 3 or 4 experiments.

The diffusiophoresis experiments were repeated under a 10 mM NaCl background concentration in both the dead-end channel and the main channel. Similar to above, 1 g/L NaPSS 70,000 or 1,000,000 Da, was initially located in the main channel. Figure 4A shows the exclusion distance over time for the NaPSS 70,000 Da, whereas Figure 4B shows the results for the NaPSS 1,000,000 Da. The presence of polyanion, as in the above cases, causes the particles to be excluded from the main channel. However, the time scale is much longer than the Milli-Q water case (Figure 4 refers to the time axis). The approximate distance after 3500 s is 349.1 ± 18.1 μm for 1 g/L 70,000 Da NaPSS and 524.4 ± 12.4 μm for 1 g/L 1,000,000 Da NaPSS. This gives an average velocity of 0.069 ± 0.006 μm/s in 70,000 Da and 0.115 ± 0.004 μm/s in 1,000,000 Da. The fluorescently labeled NaPSS (MW 70,000 Da) analysis showed that NaPSS still diffusing after 3600 s later (Supporting Information Section S5 and Figure S13). That means there are still NaPSS gradients in the dead-end channel that might influence the particle movement.

The structures of polyelectrolytes change with the addition of salt due to the interaction between the polyelectrolyte and the salt.^43,44^ NaPSS has an elongated-like structure in salt-free solution, while it becomes a coiled-like structure with increasing bulk salt concentration.^50^ The behavior of polyelectrolytes becomes more similar to that of neutral polymers with increasing salt concentration, depending on the Debye screening length and blob size.^43,51^ This is due to changes in the interaction between the charged monomers. As the salt concentration increases, the ionic density near the chain increases,^44^ and the degree of ionization of the polyelectrolyte^50^ as well as the fraction of free counterions in the solution^52^ decreases. This screens the electrostatic interaction between the charged monomers^51^ and minimizes the Coulombic and entropy-free energies.^44^

The particle dynamics are much slower with polyelectrolyte gradients in salt solutions than in the case of Milli-Q water due to the reduced electrostatic interaction in the presence of salt. As highlighted above, the polyelectrolyte behaves as a neutral polymer at high salt concentrations. To demonstrate this behavior, we performed an experiment with a PEG 1,000,000 Da gradient. The result is given in Supporting Information Figure S14. As is shown in Figure S14, the dynamics are similar to those of NaPSS in 10 mM NaCl.

### Neutral Polymer Gradient

**Figure 5:**
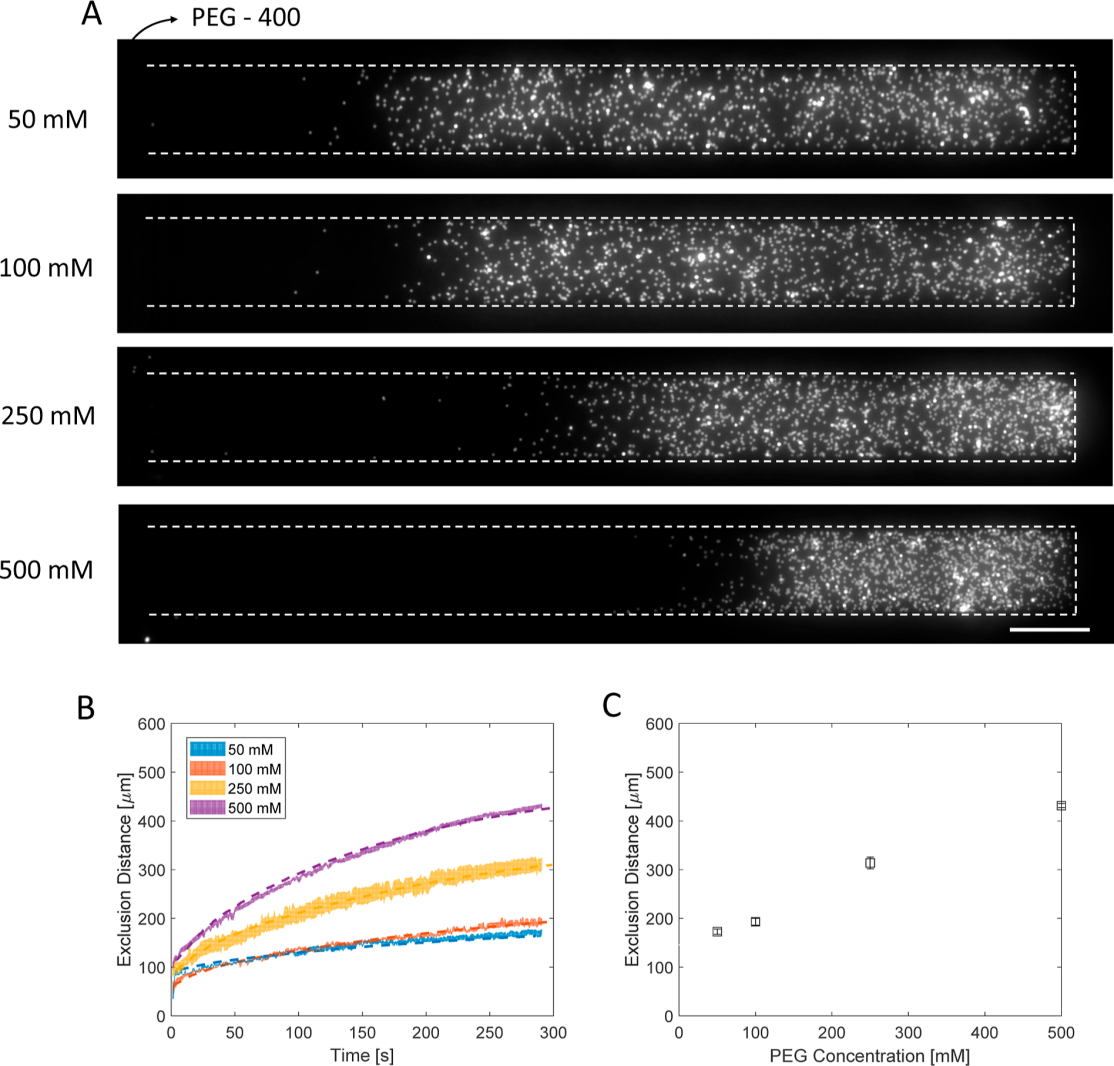
Diffusiophoretic experiment with different concentrations of PEG, where the molecular weight is kept constant (400 Da). (A) Microscope images after 290 s of the contact between the PEG solution and the particle solution in Milli-Q water. The molar concentration of PEG is indicated on the left side of microscope images: 50, 100, 250, and 500 mM. Scale bar = 50 μm. (B) Exclusion distance values in time for the experiments performed by PEG 400 Da with different concentrations are given. The color-filled areas show the standard error of 3 or 4 experiments. The dashed line given in the image shows the prediction of the simulation for a specific polymer radius. (C) Exclusion distances based on the initial main channel PEG concentration. The red line shows the linear fit. The error bar is the standard error.

As a neutral polymer, we specifically analyzed the PS particle movement under the poly(ethyl) glycol (PEG) gradient. The exclusion distance of particles was determined using different molar concentrations of PEG 400 Da (Figure 5). The main channel contains 50 mM (20 g/L), 100 mM (40 g/L), 250 mM (100 g/L), or 500 mM (200 g/L) of 400 Da PEG polymer. These polymer concentrations are below the critical concentration (c*), such that the polymer units do not overlap (see Supporting Information Figure S9). Figure 5A shows the microscope images of the dead-end channel results after 290 s of contact between the polymer solution and the particle suspension. As can also be seen from the microscope images and the exclusion distance over time (Figure 5B), the particles move away from the PEG solution into the dead-end channel, where the PEG concentration is lower. The exclusion distance at 290 s is shown with the initial polymer concentration in Figure 5C.

In the neutral polymer gradient, it is first necessary to understand the interaction between the polymer and the particle surface, whether PEG is excluded from the particle surface or adsorbed on the surface. Depending on the type of interaction between the particle surface and the polymer, particle motion can be in different directions.^6,8,10,11^ Previous observations^10,15^ and our zeta potential measurements (similar zeta potential values calculated on PEG concentrations) showed that the poly(ethylene) glycol is excluded from the particle surface mainly due to an unfavorable interaction between PEG and particle surface.^53^ Thus, eq 1 was used in our system, where the radius of the polymer, the absolute gradient of the polymer, the temperature, and the viscosity determine the diffusiophoretic velocity.

Figure 5C shows the exclusion distance at 290 s versus the initial concentration of PEG. This can be explained by eq 1 for excluded macromolecules. The exclusion length (radius of the polymer), the absolute gradient of the polymer, the temperature, and the viscosity influence the diffusiophoresis of the particles. The interplay between these parameters leads to an increase in exclusion distance in response to concentration increases. The polymer radius is not a strong function of concentration but changes with the molecular weight in the dilute regime.^17^ The absolute concentration gradient increases with the initial concentration (Supporting Information Section S7, Figure S15, S16, and S17). The viscosity for PEG 400 Da increases as well, according to our measurements (Supporting Information Figure S6). However, the increase in viscosity is less than the absolute gradient. Thus, we observed an increase in the exclusion distance based on the concentration of the neutral polymer.

We calculated the effective particle mobility (Γ_eff_) under the PEG 400 Da gradient with varying molar concentrations using the same equation as the effective diffusion coefficient  from the exclusion distance versus time analysis (Figure 5). The fitted values from fitting are given in Table 1 with 95% confidence limits. The effective particle mobility is ∼10^–10^ to 10^–11^ m^2^/s, which is similar to the PEG diffusivity in water from the Stokes–Einstein equation [*D*_pol._ = *k*_B_*T*/6πη*R*_h_ ∼ 10^–10^ to 10^–11^ m^2^/s]. Moreover, it is approximately 1–2 orders of magnitude smaller than the monovalent ion diffusivity (1–2 × 10^–9^ m^2^/s) and approximately 2–3 orders of magnitude larger than the particle diffusivity value from the Stokes–Einstein equation [*D*_p_ = *k*_B_*T*/(6πη*a*) = 4.3 × 10^–13^ m^2^/s]. When a similar determination is made for electrolyte gradients,^54^ the effective diffusion coefficient for particle values is ∼10^–9^ to 10^–11^ m^2^/s. These results underline that nonelectrolyte gradients can also be significant; however, the concentration used is much higher than in the electrolyte case. This can be explained through the driving force being the relative or absolute concentration gradients for salt or neutral polymers.

**Table 1:** Effective Particle Mobility Fitted from the Exclusion Distance Versus Time Analysis for PEG-400 Da Gradient with 50, 100, 250, and 500 mM Concentrations

*C* [mM]	Γ_eff_ [10^–11^ m^2^/s]
50	1.43 ± 0.01
100	2.68 ± 0.03
250	7.64 ± 0.08
500	17.15 ± 0.15

To have a better understanding, we simulated the system (the details can be found in the Supporting Information) by solving the unsteady-state Stokes equation with the advection–diffusion equations for the polymer and the particle in 3-D. The radius of the neutral polymer (PEG) was determined according to the agreement in exclusion distance behavior between the experiments and simulations. The simulation results over time are shown as dashed lines in Figure 5B. Moreover, Table 2 shows the fitted radii for PEG-400 from the diffusiophoresis experiments for different concentrations, as well as the hydrodynamic radius, radius of gyration, and end-to-end distance from literature. The determined radius values decrease somewhat with increasing concentration. Furthermore, they are larger than the radius of gyration and hydrodynamic radius values found in the literature. This deviation is explained below with an analysis of higher molecular weights.

**Table 2:** Fitted Radius of Neutral Polymer (PEG 400 Da) with Different Molar Concentrations from Diffusiophoresis Experiments^a^

*C* [mM]	*R*_h-lit._(17,20−22,55) [nm]	*R*_g-lit._(17,20,22,56,57) [nm]	*R*_end-to-end_(23) [nm]	*R*_fitted_ [nm]
50	0.42–0.76	0.58–0.88	1.6	1.20
100	—	—	—	1.12
250	—	—	—	1.05
500	—	—	—	1.08

a The hydrodynamic radius (*R*_h_), radius of gyration values (*R*_g_), and end-to-end distance (*R*_end-to-end_) values are taken from literature.

The properties of the aqueous PEG solution differ with the concentration of the polymer, and these variations were considered when modeling the system. (1) The viscosity of the solution increases with both the polymer concentration and polymer molecular weight. We provided the viscosity values in the Supporting Information (Figures S5 and S6). In addition, the viscosity gradients may result in particle viscophoresis.^58^ The driving force for this phoretic movement is the difference in particle diffusivity, influenced by viscosity alteration [*u*_vp_ = d*D*_p_(*x*)/d*x*].^58^ The particle diffusivity is calculated using the Stokes–Einstein equation [*D*_p_(*x*, *t*) = *k*_B_*T*/(6π*R*_h_η(*x*, *t*))]. The magnitude of this generated velocity is analyzed and provided in the Supporting Information (Figure S18). The velocity value from viscophoresis is estimated in the ∼nm/s range and is thus insignificant in our experiments. (2) The diffusivity of the polymer changes with concentration and molecular weight, as does its viscosity. This alteration is considered by using the values provided in the literature.^59^ The equations and values are provided in the Supporting Information (Figure S4). (3) The density of the solution also varies with PEG concentration and molecular weight (Figure S3), which needs to be corrected in the Stokes equation. Although we updated density values based on concentration, we still assume an incompressible fluid in the continuity equation for simplicity. (4) The size of the neutral polymer increases as the polymer’s molecular weight increases. Therefore, the molecular weight range needs to be narrow to compare the polymer’s radius with the literature accurately. To gain better insights into the molecular weight distribution, gel permeation chromatography was used to determine the distribution of molecular weights, and the polymers have a polydispersity index <1.2.

Our sensitivity analysis revealed that the exclusion distance strongly depends on the polymer radius, since the particle velocity is proportional to *R*^2^. Even a small change in the radius can significantly affect the exclusion distance. To demonstrate this phenomenon, we altered the value of the polymer radius in the simulation to determine the optimal match. We did a sensitivity analysis for PEG 6000 Da at 6.67 mM concentration, and the results are available in the Supporting Information (Figure S19).

We repeated the analysis using different molecular weights of PEG with the same molar concentrations (50 mM). The main channel was replaced by the PEG polymer with molecular weights of 2000, 3000, 4000, and 6000 Da. However, it is important to underline that the semidilute regime was reached for PEG 4000 Da (c* = 35 mM) and PEG 6000 Da (c* = 6 mM), where the polymer segments start to overlap with each other. Supporting Information Figure S9 shows the critical concentration for dilute to semidilute regions in terms of the molecular weight of the PEG.

**Figure 6:**
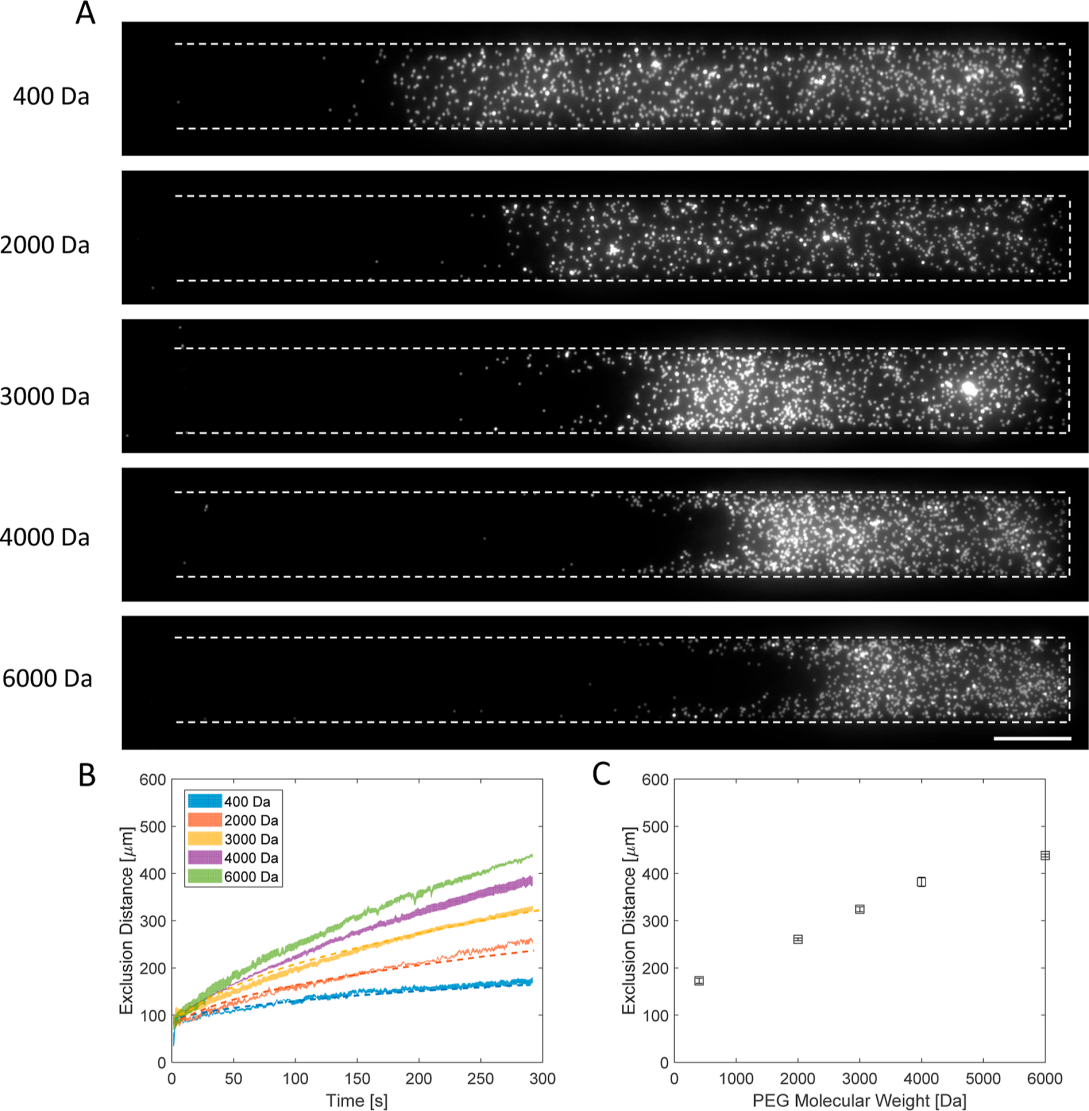
Diffusiophoretic experiment with different PEG molecular weights at a constant initial molar concentration (50 mM). (A) Microscope images 290 s after the contact between the PEG solution and the particle solution in Milli-Q water. The molecular weight of PEG is indicated on the left side of microscope images: 400, 2000, 3000, 4000, and 6000 Da. Scale bar = 50 μm. (B) Exclusion distance values in time for the experiments performed with different PEG molecular weights at the same molar concentration are given. The color-filled areas show the standard error of 3 or 4 experiments. The dashed lines show the results of simulations with the fitted polymer radius. (C) Exclusion distances at 290 s versus molecular weights of PEG are given. The error bar is the standard error.

Figure 6A presents the microscope images of the dead-end channel 290 s after contact between the polymer solution and the particle suspension. The microscope images and exclusion distance analysis over time (Figure 6B) suggest that the particles move further away from the PEG solution as the polymer’s radius increases. Table 3 also indicates that effective particle mobility is increasing with molecular weight. Even though the concentration difference is identical in each case, the absolute gradient is varied in the long term concentration distributions in the dead-end channel due to the difference in diffusivities. The concentration profile inside the dead-end channel is available in Supporting Information Figure S15. Other parameters (viscosity, diffusivity, and density) of the solution also alter with the molecular weight and concentration, so it is challenging to compare the results.

**Table 3:** Effective Particle Mobility Fitted from the Exclusion Distance Versus Time Analysis for 50 mM of PEG 400, 2000, 3000, 4000, and 6000 Da Gradient

MW [g/mol]	Γ_eff_ [10^–10^ m^2^/s]
400	0.14 ± 0.01
2000	1.15 ± 0.09
3000	1.85 ± 0.01
4000	2.53 ± 0.01
6000	3.22 ± 0.01

In the above set of experiments, there is a noticeable distinction in mass concentration between polymers with equivalent molar concentrations. To understand the impact of mass concentration on exclusion distance, we repeated the exclusion distance analysis with a 40 g/L solution for PEG 400 Da and PEG 6000 Da, where both polymers are in their dilute regime. The analysis can be found in Supporting Information Figure S20. The exclusion distance is 193.2 ± 7.1 μm for PEG 400 Da and 283.5 ± 6.2 μm for PEG 6000 Da at 290 s. The particles were excluded somewhat more when 40 g/L of PEG 6000 Da was used. This is because the values of *R* and ∇*n* are scaled differently in eq 1. The particle velocity in PEG 6000 Da is higher than PEG 400 Da (*u*_DP_ ∝ *R*^2^∇*n* = 25/15) since polymer radius (*R*) is around 5 times larger (according to Devanand and Selser^17^), and the absolute gradient is almost 15 times less in the PEG 6000 Da case. This observation is also different from the polyelectrolyte case, where the exclusion distances are similar to each other with the same mass concentration and different molecular weights.

Similar to the polyelectrolyte case, we performed experiments with a background salt present. Specifically, the dead-end channel is filled with PS particles in 10 mM NaCl solution, and the main channel concentration is exchanged with 50 mM PEG 3000 Da (150 g/L) in 10 mM NaCl background solution. The exclusion distance analysis is given in Supporting Information Figure S21. Analysis showed that the addition of a background salt does not affect the movement of the tracer particles, much unlike the polyelectrolyte case discussed previously. This underlines the fact that PEG is a neutral polymer, and its configuration and interaction are not significantly influenced by the presence of salt.

We performed 3D simulations to find out the radius of PEG for varying molecular weights. Figures 6B and S20 Supporting Information show the agreement in exclusion distance values between the experimental results and simulations. Table 4 presents the radius values obtained from the simulations and the literature values. Our radii findings are compared with the hydrodynamic radius, the radius of gyration, and the end-to-end distance found in the literature.

**Table 4:** Radius of PEG Molecular Weights of 400, 2000, 3000, and 6000 Da^a^

MW [g/mol]	*R*_h-lit._(17,20−22,55) [nm]	*R*_g-lit._(17,20,22,56,57) [nm]	*R*_end-to-end_(23) [nm]	*R*_fitted_ [nm]
400	0.42–0.76	0.58–0.88	1.6	1.1 ± 0.1
2000	1.03–1.27	1.4–2.3	4.1	1.9
3000	1.3–1.6	1.8–2.9	5.2	2.5
6000	1.92–2.65	2.6–4.5	7.8	5.4

a Hydrodynamic radius, radius of gyration values, and end-to-end distance values according to literature and radius determined from fitting the diffusiophoretic experiment.

**Figure 7:**
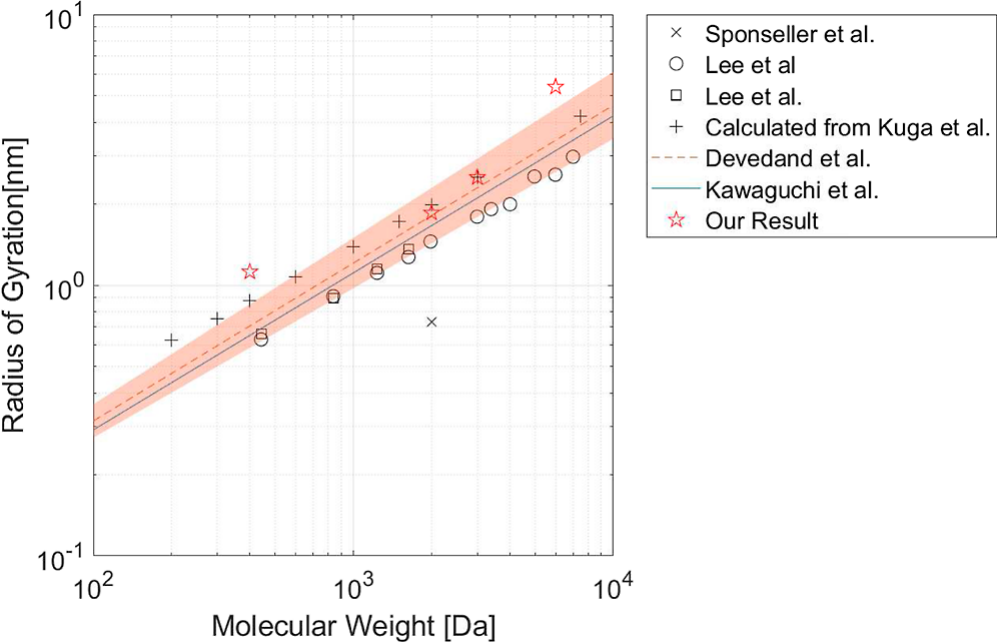
Radius of gyration values of PEG for varying molecular weights. The dashed line and its boundary are based on a relationship between the radius and molecular weight from Devanand and Selser^17^ used in the literature. A boundary shows the errors in their fittings. (*R*_g_ = 0.0215MW^0.583±0.031^). Kawaguchi et al.^56^ (−) also determined a relation between the radius of gyration and the PEG’s molecular weight (*R*_g_ = 0.02MW^0.5^). Sponseller and Blaisten-Barojas^60^ (×), Lee et al.^57^ (○), and Lee et al.^22^ (□) show the results of the computational simulations. Kuga^20^ found the hydrodynamic radius of the PEG, and by using the *R*_h_/*R*_g_ = 0.64 relation, we have calculated the radius of gyration (+).

Our fitted radius values closely align with the previously reported values for the radius of gyration, particularly PEG 2000 Da and PEG 3000 Da. Figure 7 presents the radius of gyration values obtained from the literature and the determined polymer radius for a range of molecular weights. The dashed line and shaded zone displayed in Figure 7 represent the relationship between the radius of gyration and molecular weight *R*_g_ = 0.215MW^0.583±0.031^ based on light scattering data provided by Devanand and Selser.^17^ This correlation is frequently used in the literature,^61−63^ even outside of the range of analysis, which was between 86,000 < MW < 996,000.^17^ The analysis of PEG radii is complicated when PEG has lower molecular weights due to analysis issues.^23^ It is also suggested that the behavior of PEG in water changes when the molecular weight becomes low.^22,23^

The determined radius of the polymer slightly varies with the polymer concentration (see Table 2). The concentration dependency of the radius of gyration has also been observed in previous studies for PEG 1500 Da and PEG 3400 Da.^64,65^ In the semidilute region, this decrease in blob size (mesh size) is expected since it scales with ∝*c*^–0.76^.^66,67^ The radius alterations due to varying concentrations are linked to changes in structure^65^ and repulsive intermolecular interaction.^68^ Additionally, Thiyagarajan et al.^64^ attribute the change in polymer size with concentration to excluded volume effects and possible changes in the structures of PEG. These previous works suggest a possible mechanism for slightly reducing the radius with increasing polymer concentration.

It is important to note that the polymer solution contains a distribution of molecular weights rather than a single molecular weight. Supporting Information Figure S7 and Table S3 give the molecular weight distribution of the polymer as measured by gel permeation chromatography. The polydispersity index of the polymers (PEG 400, 2000, 3000, and 4000 Da) is <1.1. However, for PEG 6000 Da, the polydispersity is somewhat higher than that for the other molecular weights. In addition, the *k*_D_ parameter (see Supporting Information) used to determine the diffusivity of PEG 6000 Da is approximated using a similar approach as in Peppin.^69^ This may contribute to the higher polymer radius obtained for PEG 6000 Da compared to literature values for the radius of gyration.

Overall, our study indicated a consensus between the calculated radius of gyration and the literature values for PEG 2000 Da and PEG 3000 Da. In the case of PEG 400 Da and PEG 6000 Da, the determined polymer radii deviate somewhat from the literature radii of gyration but are still in the same order of magnitude. These deviations might be associated with the structural change in low concentration (for PEG 400 Da) and the variation in molecular weight distribution or possible wrong estimation of diffusivity (for PEG 6000 Da). In general, it can be inferred that the dead-end channel system is effective in the analysis of the polymer’s radius of gyration (similar to zeta potential determination^38^).

### Nanoparticle Gradient

For all the neutral polymer analyses above, we assume an effective hard-sphere model for the neutral polymer. This model is used in the literature^69−71^ to describe the polymer structure in a solution. We considered the polyelectrolyte case, where the polymers are charged, and observed the PS microparticle movement under the polyelectrolyte gradient. On the other hand, the nanoparticles, which are spherical in shape (similar to the hard-sphere model), are charged in water, providing colloidal stability and excluding them from the PS microparticle surface. These nanoparticles can be used to create an osmotic imbalance in the system (to drive the PS particles).^72^ Therefore, we now investigate how hard-sphere nanoparticle gradients affect the diffusiophoresis of microparticles.

**Figure 8:**
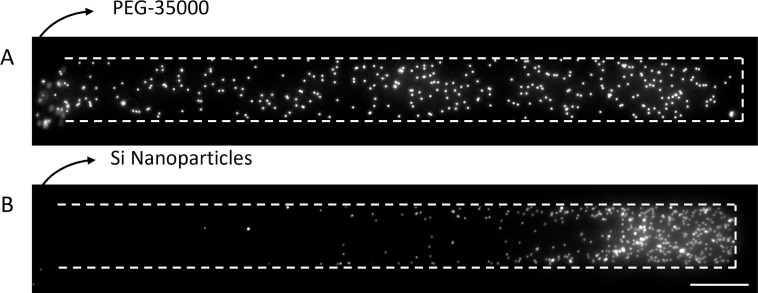
Comparison between gradients formed by neutral molecules (PEG) and Si-particles. The molar concentration in the main channel is (A) 0.112 mM of PEG 35,000 Da and (B) 0.022 mM of silica nanoparticles (=ϕ_1_ = 0.039 = 7.5 wt %). The approximate *R*_g_ of the PEG polymer is 6.9–13.3 nm based on the Devanand and Selser,^17^ and the radius of the silica nanoparticle is also 8.9 ± 0.9 nm. The microscope images were taken 300 s after contact. Scale bar = 50 μm.

We performed an experiment using a neutral polymer (PEG MW 35,000 Da) of approximately *R*_g_ as 10 nm (6.9–13.3 nm based on Devanand and Selser^17^), and silica nanoparticles with radius *a* of 8.9 ± 0.9 nm (based on DLS measurements). The molar concentration of the silica particles was 0.022 mM, and SEM images of the particle are given in Supporting Information Figure S22. The zeta potential of these particles was determined as ∼−30 mV at pH = 5.6–5.8. For PEG experiments, the initial molar concentration of polymer was 0.112 mM in the main channel. Figure 8 shows the microscope images after 300 s of contact. The upper part of Figure 8 shows the microscope image with the PEG 35,000 Da, whereas the lower part shows the experiment with the silica particles. The microscope images clearly indicate that the particle exclusion is very different (Figure 8). The exclusion distance observed with silica particles is far greater than that observed with PEG 35,000 Da, even though the polymer molar concentration is 5 times higher. This is despite the particle and polymer having approximately the same radius and both being excluded from the microparticle surface. The main difference is that the PEG is an uncharged polymer, whereas silica particles possess surface charge due to their surface hydroxide group dissociation. In addition, silica particles do not display any water uptake, whereas PEG polymers do.

**Figure 9:**
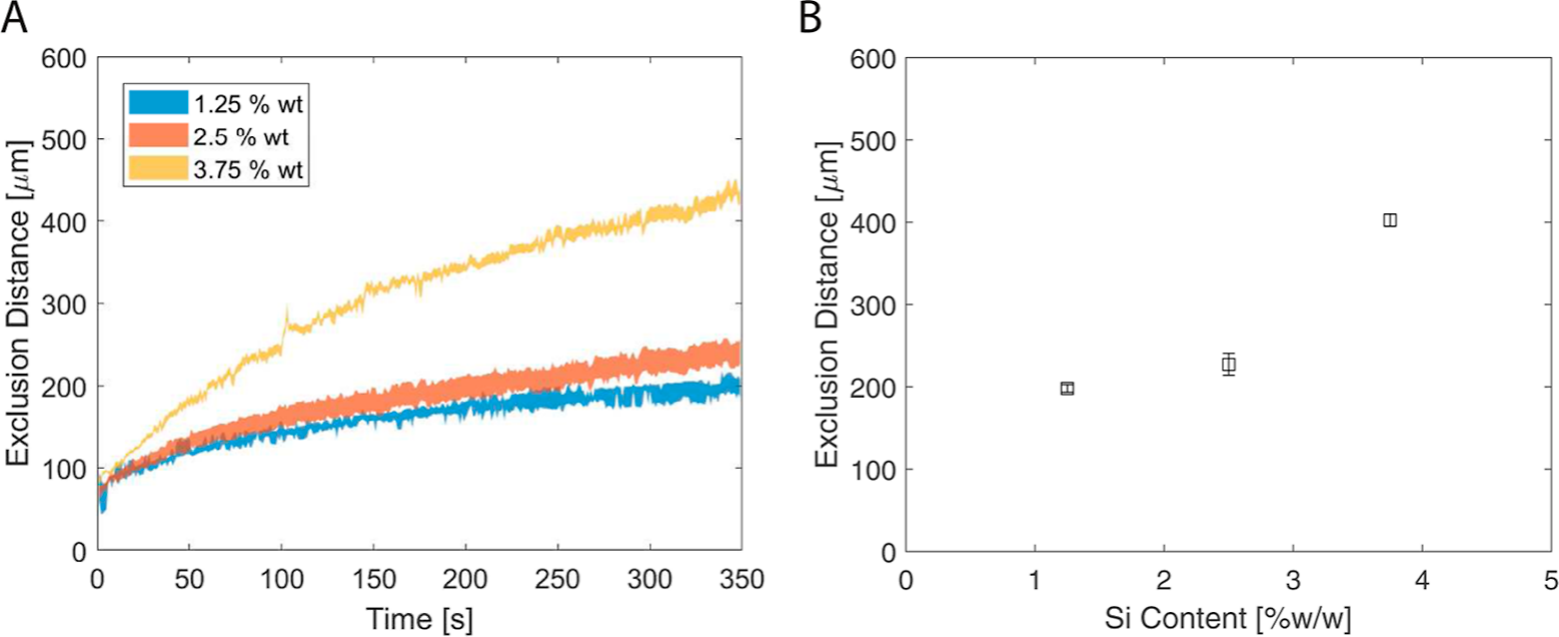
Change in exclusion distance with silica content (% w/w). (A) Excluded distance values in time for the experiments performed by silica particles with different weight fractions are given. The color-filled areas show the standard error of 3 experiments. (1.25 wt % ≡ ϕ_1_ = 0.0063, 2.5 wt % ≡ ϕ_1_ = 0.013, 3.75 wt % ≡ ϕ_1_ = 0.019) (B) Excluded distance values at *t* = 350 s for different initial silica contents. The error bar is the standard error.

We also conducted experiments with varying initial volume fractions (concentrations) of the silica particles in the main channel. Figure 9A shows the exclusion distance values for different weight fractions over time. The exclusion of particles increases with higher concentrations as the driving force is stronger (Figure 9B). Based on our results, if lower initial volume fractions than 0.0063 (1.25 wt %) were used, a smaller exclusion zone size would be expected, and this may be challenging to quantify experimentally.

**Figure 10:**
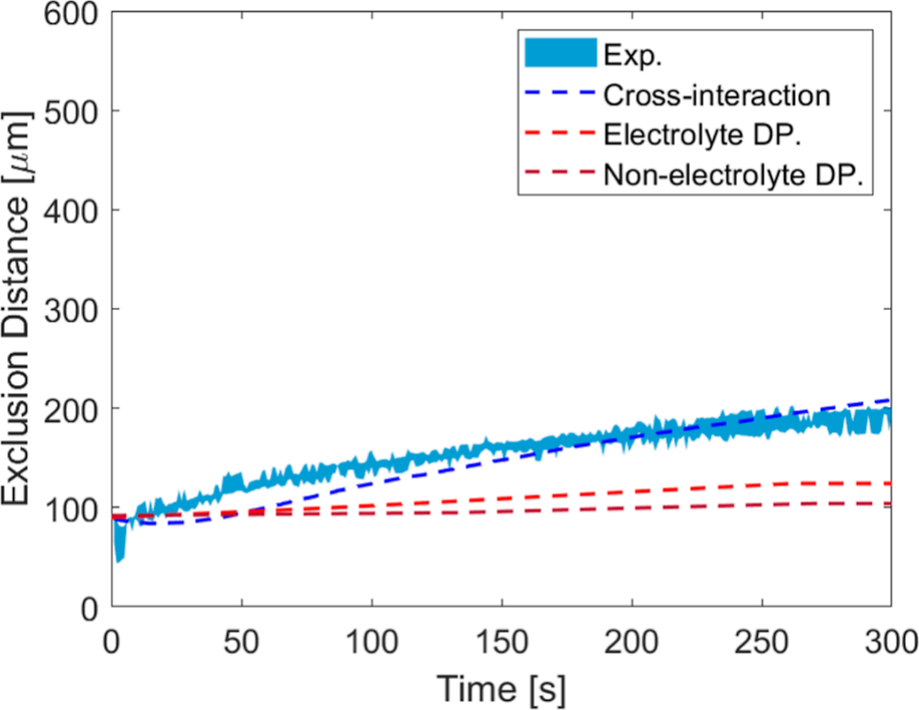
Different approaches to describe tracer movement in nanoparticle gradient. The experimental result shows the case when the initial silica weight fraction in the main channel is 1.25 wt % ≡ ϕ_1_ = 0.0063. The simulation details are in the Supporting Information Section S1.4.

To understand the behavior of the silica particles better, we conducted simulations using three different approaches. The details of the simulations are given in the Supporting Information Section S1.4. (1) Silica nanoparticles are excluded from the tracer particle surface (in the dead-end channel) and behave in a nonelectrolyte diffusiophoretic motion,^14^ similar to the PEG observation above. Equation 1 was used for the analysis. (2) Tracer particles are experiencing electrolyte-diffusiophoresis.^73^ We assumed that silica nanoparticles are quite small compared to PS microparticle and might act as a salt as one big molecule with a dissociate group. Equation was used as the diffusiophoretic velocity in this case. (3) Finally, the cross-interaction between the different particle sizes was considered by assuming the hard sphere model.^74^Equations S11 and S12 were used in this system in 1-D (only dead-end channel). The results of the simulations and the experimental value for 1.25 wt % case are shown in Figure 10. The experimental observations are better described by the cross-interaction between the particles.

The cross-interaction theory of binary colloidal mixtures is valid for dilute mixture systems.^74^ This theory is also used to describe the other silica fractions given in Figure 9. The results are given in Supporting Information Figure S23. Based on the ratio of particle radii used in this work, there is no influence of the initial volume fraction of the larger particles on the exclusion distance observed as consistent with the cross-interaction model (Supporting Information Figure S24).

Equations S11 and S12 depend highly on the particle size ratio (α = *R*_PS_/*R*_silica_). To illustrate this, we performed the same experiment with silica particles of 15 nm radius. A slight decrease in the exclusion of PS particles is observed at this lower value of α, in agreement with the simulation results and examination of the differential equations that form the cross-interaction model we used (Supporting Information Figures S25 and S26).

## Conclusions

This paper analyzes the behavior of PS particles in a dead-end channel under varying the gradients of polyelectrolytes, neutral polymers, and silica particles. First, we observed the behavior of the particles under NaPSS polyelectrolyte gradients. In contrast to diffusiophoresis in the NaCl gradient, particles in a dead-end channel exhibited a completely opposite directionality in NaPSS gradients due to the exclusion behavior of NaPSS. The exclusion distance of PS microparticles remained almost constant at the same mass concentrations but at different molecular weights, while it increased in the PEG case with higher molecular weights. Moreover, we observed different time scales when salt is present in the bulk of the NaPSS solution. The time scale approaches that of the PEG gradient at a similar molecular weight. These show that the polyelectrolyte behaves as a neutral polymer when salt is present in the bulk. This is due to the screened electrostatic interaction.

Second, the movement of the particles was studied in the gradients of a neutral macromolecule, PEG, which is excluded from the particle surface. As the concentration of the polymer (at the same molecular weight) and the molecular weight (at the same concentration) increase, the exclusion distance of the particles also increases. The exclusion length, which was previously associated with the polymer radius, of the PEG polymer was investigated through simulations. The values obtained from the simulations were similar to the values of the radius of gyration found in the literature.

Finally, we compared the gradient type with the silica nanoparticles to that of the PEG molecules, as the PEG polymer is assumed to be a hard sphere in the simulation. Our results indicate that the exclusion distance is higher in silica nanoparticle gradients due to the fact that silica nanoparticles are charged, whereas PEG is not. Moreover, the theory of the cross-interaction between the PS microparticle and the silica nanoparticle is in better agreement with the experimental values compared to the diffusiophoretic behavior of the particle (nonelectrolyte or electrolyte). Overall, we found that excluded solute gradients can exhibit different diffusiophoretic behaviors depending on the nature of the solute. The concept presented here is valid for the case where the macromolecule is excluded from the particle surface and can be applied to other macromolecules, such as proteins. Moreover, this approach given here could be used to analyze the concentrations, molecular weights, or radii of these excluded macromolecules.

## Supporting Information Available

Simulation’s theoretical background, equations, and initial/boundary conditions; values for varying molecular weights of aqueous PEG diffusivity, viscosity, and density; hydrodynamic radius, critical concentration, and analysis of molecular weight distribution; normalized concentration and absolute gradient behavior of PEG 400 Da with different concentrations; and further experiments concerning equivalent PEG mass concentrations, silica particle gradients, and NaPSS without a cleaning step (PDF)
