# Supplementary material for: Diffusiophoresis in Polymer and Nanoparticle Gradients
Source: J Phys Chem B. 2024 Jun 5;128(24):5874–87. doi: 10.1021/acs.jpcb.4c00985 (PMC11194826; doi:10.1021/acs.jpcb.4c00985)
Supplement: Supplementary file 1 — jp4c00985_si_001.pdf [file jp4c00985_si_001.pdf]

Supporting Information: Diffusiophoresis in Polymer and Nanoparticle Gradients

Burak Akdeniz, Jeffery A. Wood, and Rob G. H. Lammertink*

Soft Matter, Fluidics and Interfaces, University of Twente, MESA+ Institute for Nanotechnology, P.O. Box 217, 7500 AE Enschede, The Netherlands

E-mail: r.g.h.lammertink@utwente.nl

Phone: +31 (0)534892063

Contents

S1 Theoretical Background and Simulations 4

S1.1 Theoretical Diffusiophoretic Movement under Non-Electrolyte Gradient . . . 4

S1.2 PEG Gradient Simulations with Boundary & Initial Conditions 5

S1.3 Asymmetric Electrolyte Diffusiophoresis 8

S1.4 Silica Particle Gradient Simulations with Boundary & Initial Conditions . . 8

S1.4.1 Non-Electrolyte Diffusiophoresis 9

S1.4.2 Electrolyte Diffusiophoresis 10

S1.4.3 Cross-Interaction 10

S2 Exclusion Distance Determination 11

S3 Proporties of the PEG 11

S3.1 Density, viscosity and the diffusivity of the aqueous PEG 12

S3.1.1 Density of the PEG 12

S3.1.2 Diffusivity of the PEG 12
S3.1.3 Reduced Viscosity of the PEG 14
S3.2 Molecular Weight Distribution 16
S3.3 Hydrodynamic Radius 17
S3.4 Critical Concentration 18

S4 Dialysis of the Polyelectrolyte Solutions 19
S4.1 Diffusiophoresis Results of the Polyelectrolytes without Dialysis 19
S4.2 Ion Chromatography Results of Permeate of Dialysis 21

S5 Labelled NaPSS Analysis 22

S6 Analysis of 1 g/L of 1,000,000 Da PEG 23

S7 Simulations with PEG Gradient 24
S7.1 PEG Concentration Profile Inside Dead-end Channel 24
S7.2 PEG Absolute Gradient Inside Dead-end Channel 25
S7.3 Viscophoretic Velocity 27
S7.4 Sensitivity Analysis of Simulations 28

S8 Experiments with the Same Mass Concentration of PEG 29

S9 Experiments with Background Salt - PEG 30

S10 SEM Images of Silica Particles 31

S11 Simulation Results of Silica Nanoparticle Gradient for Different Gradients 32

S12 Simulation Results of Silica Nanoparticle Gradients with Varied PS Fractions 33

S13 Simulation and Experimental Results of Smaller Silica Nanoparticle Gradients **34**

References **36**

S1 Theoretical Background and Simulations

We give the theoretical background for the non-electrolyte diffusiophoresis. Moreover, the details of the simulations are given with equations and the initial/boundary conditions. Simulations mentioned in this section were performed using the finite element method in COMSOL Multiphysics 6.0. The mesh independence was checked by performing simulations in lower meshes and controlled via concentration profiles.

S1.1 Theoretical Diffusiophoretic Movement under Non-Electrolyte Gradient

The particles performed diffusiophoretic movement under a non-electrolyte gradient. The interaction between a solute and a particle surface (represented by a potential energy Φ) determines whether the particles move higher or lower concentration sides.[1] The origin of the interaction can be exclusion volume effects, dipole, or van der Waals forces.[1–3] The diffusiophoretic analysis has been presented previously.[3–5] The diffusiophoretic velocity[3,4,6] of a particle for a non-electrolyte solute is given below.

$$u_{DP} = \frac{k_B T}{\eta} K L^* \frac{dc}{dx}, \tag{1}$$

where k_B is Boltzman constant, T is the medium absolute temperature, η is viscosity. The potential energy is represented in the terms K[m] and L^*[m]. KL^* can be approximated under certain assumptions that enable the determination Φ. When the interaction is steric (entropic), where the solute is not adsorbing but excluding from the surface, the equation is reduced to eq. 2.

$$KL^* = -\frac{R^2}{2}, \tag{2}$$

where the radius of the solute is, in our case, the radius of polymer (R), with this

assumption, the diffusiophoretic velocity of the non-electrolyte where the solute is excluded from the surface becomes the following equation.

$$u_{DP} = -\frac{k_B T}{2\eta} R^2 \frac{dc}{dx}, \tag{3}$$

When the solute is excluded from the particle surface, particles start to move towards the low-concentration side,[6] which we also observed in the experiments (see Figure 3). Below, we underline the equations that we solved in the simulation.

S1.2 PEG Gradient Simulations with Boundary & Initial Conditions

The movement of the fluid is captured with the Navier-Stokes equation. The inertia term was ignored from the Navier-Stokes equation because of the low Reynolds number ($Re \ll 1$). Unsteady Stokes (eq. 4) and fluid continuity (eq. 5) equations for incompressible fluids were used to describe the fluid flow inside the dead-end channel.

$$\rho \frac{\partial \boldsymbol{u}}{\partial t} = -\nabla p + \eta \nabla^2 \boldsymbol{u}, \tag{4}$$

$$\nabla \cdot \boldsymbol{u} = 0, \tag{5}$$

where ρ is the fluid density, η is the fluid viscosity, and p is the pressure. However, it should be noted that $\rho(x,t)$ and $\eta(x,t)$ are the function of the space and the time. In the simulations, we have altered the values of $\rho(x,t)$ and $\eta(x,t)$ depending on concentration. However, we assumed incompressible fluid in the continuity equation (eq. 5) due to simplicity. For densities of PEG at different molecular weights, Ninni et al.[7] provided a linear fit of the experimental results of Kirincic et al.[8] The values for this linear fit are used, and the results are given below.

Table S1: Density of PEG in aqueous solutions at 25°C $\rho = a + bw$. The densities are in g/cm^{-3}. w shows the weight fraction of the PEG. The a and b values are taken from Ninni et al.[7] The experimental value belongs to Kirincic et al.[8]

Mw [g/mol]	a	b
400	0.99611	0.16985
2000	0.99577	0.17862
3000	0.99574	0.17943
6000	0.99575	0.18059

The viscosity values for the PEG 400 Da and PEG 2000 Da with our measurements are given below. We have used the same approach with Peppin,[9] where a hard-sphere model for PEG was used. They used the data of Vergana et al.[10] to fit the equation $\eta = \eta_0 e^{k_\eta \phi_1}$, where η_0 is the solvent viscosity, k_η is the viscosity coefficient, and ϕ_1 is the volume fraction of PEG. The coefficient values are discussed below.

Since we stopped the flow after contacting two fluids, we set the pressure at the outlet point to 0 Pa as an arbitrary value. We assumed that the PDMS is also excluded from the particle surface. This creates a slip velocity at the dead-end channel surface, which leads to a recirculation flow similar to the case with the salt.[11–20] Thus, we applied a wall slip velocity, which we defined as the diffusio-osmotic velocity at all dead-end channel walls:

$$\boldsymbol{u}_{DO} = u_{slip} = -u_{DP} = \frac{k_B T}{2\eta} R^2 \frac{dc}{dx}, \tag{6}$$

The polymer concentration was determined with the advection-diffusion equation, where we assumed PEG polymer as an effective hard-sphere model.[9] Here, we need to underline that the polymer diffusivity, viscosity, and density change according to the polymer concentration and the molecular weight.

$$\frac{\partial c}{\partial t} + \nabla \cdot (\boldsymbol{u} c) = \nabla (D \nabla c), \tag{7}$$

where D is the diffusion coefficient and changes with polymer concentration.[21] We used $D = D_0(1 + k_D \phi_{PEG})$, and $\eta = \eta_0 exp(k_e ta \phi_{PEG})$ and the values are given in Table S2.

The coefficients of PEG 400 Da and PEG 2000 Da are taken from Peppin et al.[9] Other calculated according to the given procedure in Peppin et al.[9] These coefficients were used in the simulation to consider diffusivity and viscosity change.

D_0 is the self-diffusion coefficient of PEG at infinite dilution, which is calculated by using the Stokes-Einstein equation ($D_0 = k_b T/6\pi\eta_s R_h$). R_h is the hydrodynamic radius of PEG, which was calculated by $R_h = 0.145 M_w^{0.571\pm0.009}$ in [Å][22] and can be found in detail in the next section.

Table S2: The coefficients used in the simulation.

Mw [g/mol]	D_0 x 10^{-10} [m^2/s]	k_η	k_π	k_D
400	4.4	5.7	1.85	-0.60
2000	2.0	5.2	1.98	0.06
3000	1.6	4.2	1.81	0.48
6000	1.0	4.0	4.01	4.99

The walls of the dead-end channel contribute to the flow within the channel through diffusio-osmotic flow ($u_{DO} = -u_{DP}$). The concentration of PEG inside the dead-end channel starts at 0 mM, while the main channel concentration is set to 50-100-250-500 mM for PEG-400, 50 mM for PEG 2000 and PEG 3000, and 6.67 mM for PEG 6000.

The particle dynamics inside the dead-end channel were estimated using the advection-diffusion equation. A similar approach is taken for the case of electrolyte diffusiophoresis.[12–20] The convection term includes the diffusiophoretic velocity of the particles combined with the fluid flow generated by diffusio-osmosis inside the dead-end channel. We did not include other interactions, particle-particle or particle-wall interactions.

$$\frac{\partial c_p}{\partial t} + \nabla \cdot (\boldsymbol{u_p} c_p) = \nabla(D_p \nabla c_p), \tag{8}$$

where D_p is the particle diffusion coefficient which is estimated by the Stokes-Einstein equation ($D_p = k_B T/(6\pi\eta a)$). $\boldsymbol{u_p}$ is obtained as the summation of particle diffusiophoresis ($\boldsymbol{u_p} = u_{DP} + u$). u_{DP} is from eq. 3 and the fluid flow ($\boldsymbol{u}$ is from eq. 4 and eq. 5). The

particle concentration inside the dead-end channel is 1, where the experiment points started. In other locations, the particle concentration is set to 0.

S1.3 Asymmetric Electrolyte Diffusiophoresis

For the asymmetric valency electrolytes, diffusiophoretic velocity description changes. The velocity description is given for two different cases depending on the zeta potential of the particle or surface. The derivation can be found in.[23] The derivation was done in dimensionless numbers. Thus, we have used the capital notation here. Below, we rewrite the equation for two cases. When the dimensionless zeta potential is smaller the one ($|\Psi_d = \zeta/(k_B T/e)| << 1$), the equation becomes,

$$U_{DP} = -\left(-\beta\Psi_D - \frac{\Psi_D^2}{8} - (Z_+ + Z_-)\frac{\Psi_D^3}{216}\right)\nabla \ln C, \tag{9}$$

where $\beta = (D_+ - D_-)/(Z_+ D_+ - Z_- D_-)$, and Z is the valance ($Z_+$ for cation, Z_- for anion). The equation becomes in the following form when the zeta potential is negative and higher than $\sim$ 25 mV.

$$U_{DP} = -\left(-\beta\Psi_D + \left(\frac{\Psi_D + \Psi_l}{Z_+} - \frac{2(exp[Z_+(\Psi_D + \Psi_l)/2] - 1)}{Z_+^2}\right)\right)\nabla \ln C, \tag{10}$$

where Ψ_l is estimates as $= Z_+^{-1}(4ln2 - 2)$. The first term in both equations is defined as the electrophoretic term, and the rest is the chemiphoretic term. Both equations are solved for varied zeta potential and Z_+ = 1 and Z_- = -337, and β = 0.039. The results are shown below.

S1.4 Silica Particle Gradient Simulations with Boundary & Initial Conditions

For the simulation of the silica particles, we performed three separate approaches.

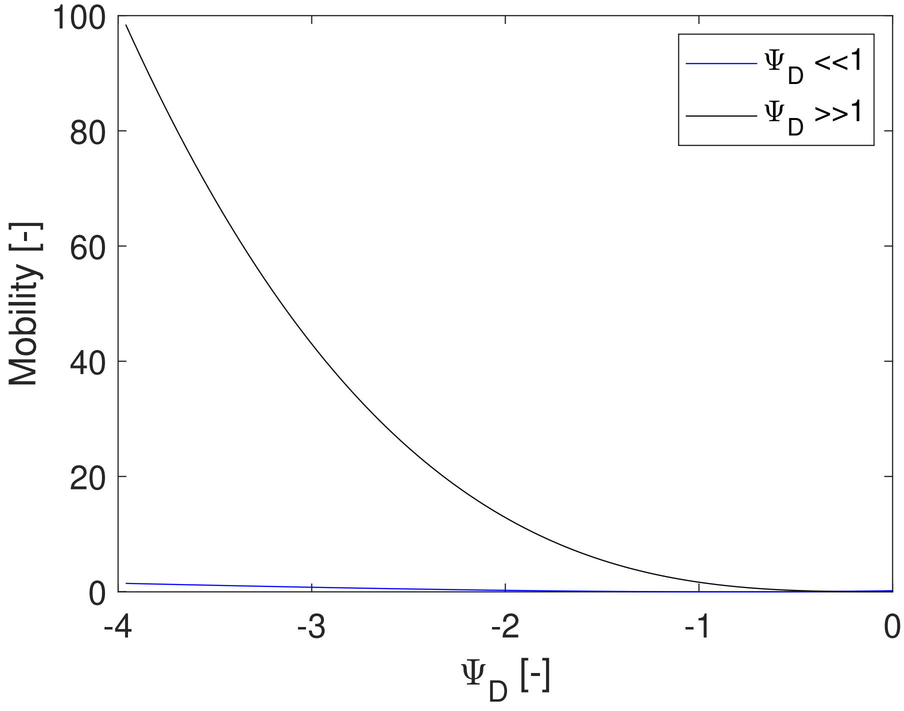

Figure S1: The diffusiophoretic mobility for asymmetric valance for polyelectrolyte case.

S1.4.1 Non-Electrolyte Diffusiophoresis

In this method, we have repeated the above procedure. Since the silica particles are excluded from the surface of the PS particles, the above approach for the PEG seems to be applicable also for the silica particles. In addition, the non-electrolyte diffusiophoresis approach was used for the stratification in films.[24] However, we need to underline that Staffeld et al.[25] used exclusion radius R for charged particles, as summing radius with Debye length $(R+2*\kappa^{-1})$. In our case, we used MilliQ water in particle in both sides of the channel. The ion source is untreated ions in MilliQ and the dissolved carbon dioxide (bicarbonate ions). We have solved the equations by taking $R \approx 10$ nm (the measured value in DLS was 8.9 ± 0.9 nm) and $R + 2 * \kappa^{-1} \approx 300$ nm. In the main text, we showed the results of $R \approx 10$ nm. The simulations with the other setting is not converge in above settings.

Additionally, the viscosity was corrected according to Einstein's effective viscosity formula $(\eta = \eta_0(1+2.5\phi))$, whereas the density is kept constant. The boundary and initial conditions

are the same as above.

S1.4.2 Electrolyte Diffusiophoresis

In this method, we used an approach similar to that described above. We assumed that the silica nanoparticles are quite small and act as an asymmetric salt, and the tracer particles were assumed to undergo electrolyte diffusiophoresis. We changed the description of $\boldsymbol{u_p}$ in eq. 8 as the description of u_{DP} is changed. For the description of diffusiophoresis we have used the diffusiophoretic description in electrolytes,[23] given in eq. 10.

In the equation, ζ is the zeta potential of the PS particle (assumed to be - 80 mV[13]), β is the diffusivity difference of the ions ($\beta = (D_+ - D_-)(Z_+D_+ - Z_-D_-)$, here we assume D_+ as proton diffusivity = 9.3 · 10^{-9} m^2/s, and D_- as the particle diffuvisity calculated from the Stokes-Einstein equation) and Z is the valency of the solute 1 for the proton and (Z_-) determined as to be -16. For the calculation, the same approach was used in.[26]

Additionally, the viscosity was corrected according to Einstein's effective viscosity formula ($\eta = \eta_0(1+2.5\phi)$), whereas the density is kept constant. The boundary and initial conditions are the same as above.

S1.4.3 Cross-Interaction

In the final method (ZJD model), we did not use any of the above equations. The cross-interaction between the different sized particles was considered by assuming the hard sphere model.[27] The equations 11 and 12 have been used in 1-D (only dead-end channel). The theory is valid for dilute mixture systems.[27]

The simulations were performed considering only the dead-end channel - 1D by solving eq. 11 and eq. 12.

$$\frac{\partial \phi_1}{\partial t} = D_1 \frac{\partial}{\partial x}\left[(1+8\phi_1)\frac{\partial \phi_1}{\partial x} + \left(1+\frac{1}{\alpha}\right)^3 \phi_1 \frac{\partial \phi_2}{\partial x}\right], \tag{11}$$

$$\frac{\partial \phi_2}{\partial t} = D_2 \frac{\partial}{\partial x}\left[(1+\alpha)^3 \phi_2 \frac{\partial \phi_1}{\partial x} + (1+8\phi_2)\frac{\partial \phi_2}{\partial x}\right], \tag{12}$$

where notation 1 was used for the silica particles and 2 for the PS particles. D represents the diffusion coefficients and was determined using the Stokes-Einstein equation. α is the fraction of the radius $= R_2/R_1$. ϕ is the volume fraction.

The viscosity was corrected according to Einstein's effective viscosity formula ($\eta = \eta_0(1+2.5\phi)$). The simulation was performed for the different volume fractions of the particles (ϕ_1). We assumed $\phi_1 = 0.0063$, 0.013 and 0.019 and $\phi_2 = 0$ at the inlet of the dead-end channel. We also assumed no flux boundary condition ($\frac{\partial \phi}{\partial x} = 0$) at the end of the dead-end channel (@ x = 600 µm). The initial volume fraction was also assumed to be $\phi_1 = 0$ and $\phi_2 = 0.00087$ $\approx$ 500 particles in the dead-end channel. The results are given in the main text and below.

S2 Exclusion Distance Determination

Figure S2 shows the exclusion distance determination graphically. The images were taken in the camera at 1 or 10 frames per second. For 10 fps, the frame rate was reduced to 1 fps. The normalized grey value was determined in the x direction. The minimum and maximum normalized grey values were determined. The end of the minimum value and the beginning of the maximum value were averaged to get the middle point as shown in the figure. This distance is called the exclusion distance.

S3 Proporties of the PEG

Density, diffusivity, and viscosity values used in the simulations are listed below. Furthermore, the molecular weight distribution, hydrodynamic radius values, and critical concentration values are also given below.

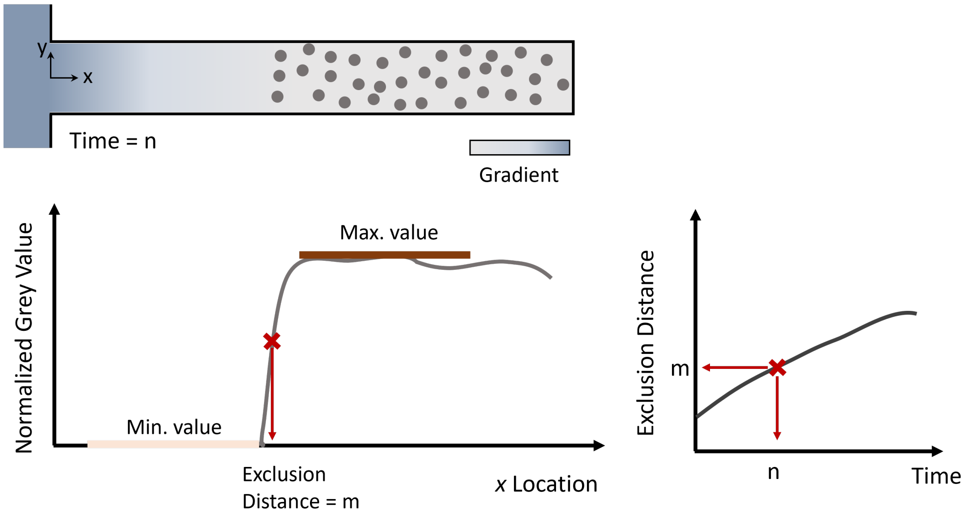

Figure S2: The graphical illustration of exclusion distance determination at time n.

S3.1 Density, viscosity and the diffusivity of the aqueous PEG

Here, we showed the concentration dependence of density, viscosity, and diffusivity of the aqueous PEG. Since diffusiphoresis is a concentration-dependent process, here we underline the parameters that vary with the concentration of the PEG.

S3.1.1 Density of the PEG

The solution density varies with the PEG concentration in water. The density of the solution increases as the PEG concentration rises, and this increase is more pronounced with higher molecular weights. Figure S3 shows the normalized density of aqueous PEG solution at 25 °C. We have used the fitting results of Ninni et al.[7] ($\rho = a + bw$). The a and b values are given in Table S1. Ninni et al.[7] used the experimental data of Kirincic et al.[8]

S3.1.2 Diffusivity of the PEG

The diffusivity of the polymer in water varies depending on the concentration and molecular weight of PEG. In our study, we used a hard-sphere model presented in Peppin's research,[9] which employed k_D (refer to Table S2) to determine the PEG diffusivity. The study also showcased the consistent outcomes of experimental values from Vergara et al.[21] The diffusiv-

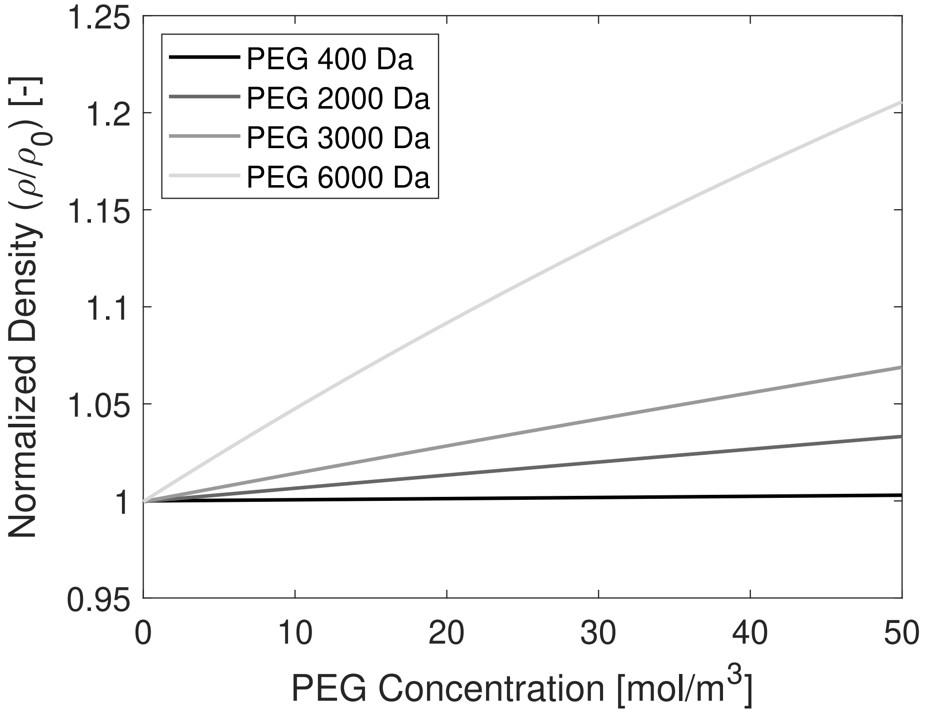

Figure S3: The normalized density of aqueous PEG solution. PEG 400 Da, 2000 Da, 3000 Da, and 6000 Da are the molecular weights.

ity was normalized by dividing it with D_0, which was calculated through the Stokes-Einstein formula employing the polymer's hydrodynamic radius. The normalized diffusivity values are given in Figure S4.

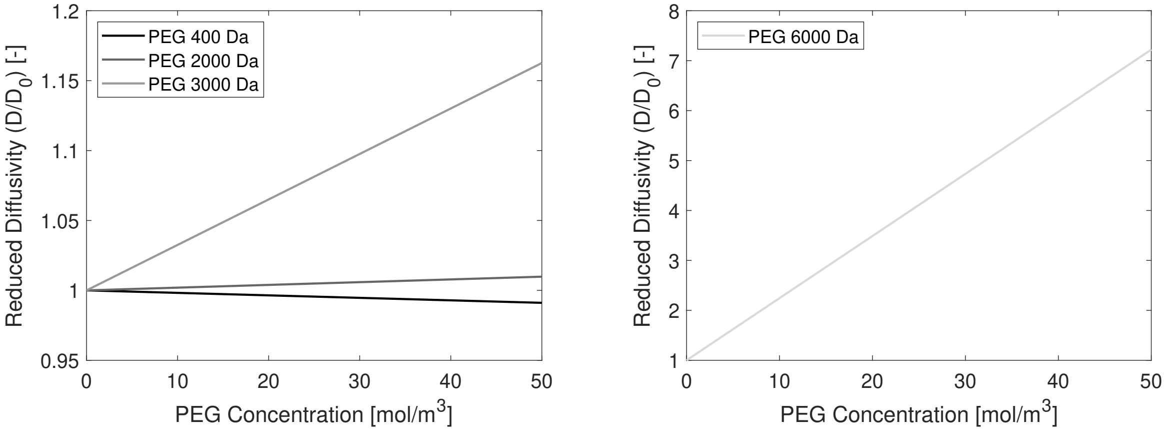

Figure S4: The diffusivity of PEG polymer in water at 25°C was given, with molecular weights of various ranges and polymer concentrations.

S3.1.3 Reduced Viscosity of the PEG

The reduced viscosity of the polymer in water varies depending on the concentration and molecular weight of PEG. In our study, we used a hard-sphere model presented in Peppin's research,[9] which employed k_η (refer to Table S2) to determine the PEG reduced viscosity. The study also showcased the consistent outcomes of experimental values from Vergara et al.[10] and Albright et al.[28] For PEG 400 Da, we have also measured the polymer viscosity given in Figure S6.

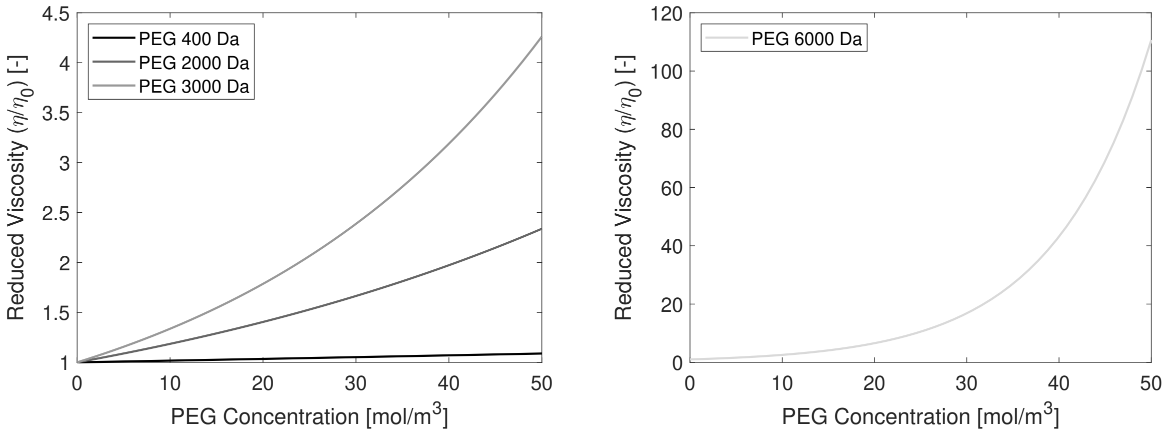

Figure S5: Relative viscosity values of PEG 400 Da, PEG 2000 Da, PEG 3000 Da, and PEG 6000 Da polymer at 25°C over polymer molar concentration.

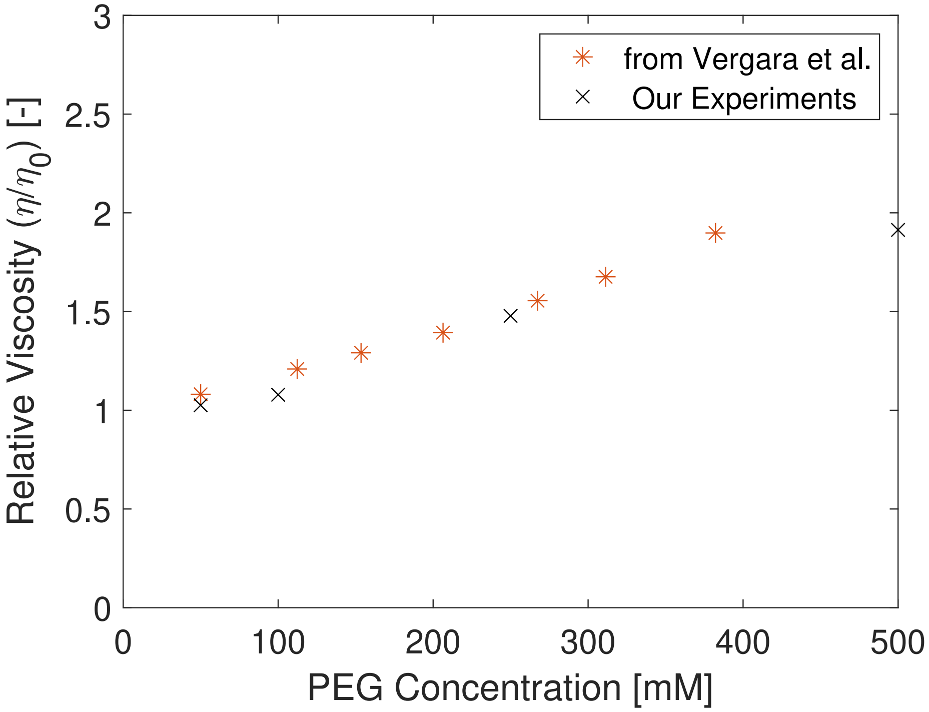

Figure S6: Relative viscosity values of PEG 400 Da in different concentration values at 25°C. The cross symbols ($\times$) show our results, and the $*$ symbols show the values from the literature.[10] In our results, we measured the viscosity of the PEG at shear rates of 1 - 1000 s^{-1}. The polymer solution at this concentration behaves as Newtonian fluid (viscosity values did not change with shear rate). The represented results are shown in cross symbols ($\times$) by averaging the values.

S3.2 Molecular Weight Distribution

Gel permeation chromatography (GPC) was employed to evaluate the molecular weight distribution of the neutral polymer (PEG). The outcomes are presented in Table S3, and in Figure S7.

Table S3: Number and weight averaged molecular weights are given based on initial PEG molecular weight. The polydispersity index (PDI) of PEGs is determined by $\bar{M}_{\mathrm{w}}/\bar{M}_{\mathrm{n}}$

.

PEG MW [Da]	$\bar{M}_{\mathrm{n}}$ [Da]	$\bar{M}_{\mathrm{w}}$ [Da]	PDI
400	380	408	1.07
2000	1762	1827	1.04
3000	2829	2962	1.05
4000	3736	3936	1.05
6000	4937	5847	1.18

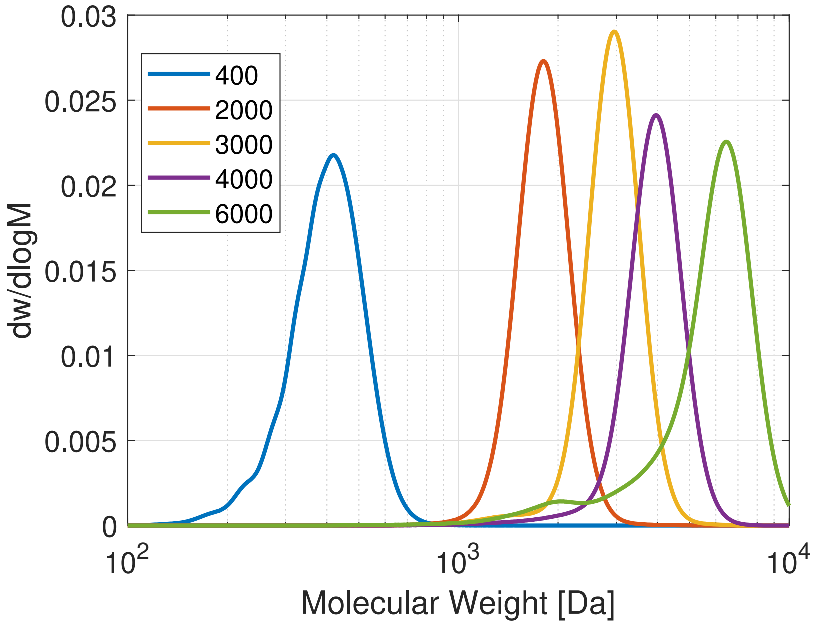

Figure S7: GPC results for the different molecular weights of the PEG. The molecular weight distribution of the PEGs are given in the figure.

S3.3 Hydrodynamic Radius

The hydrodynamic radius of PEG is obtained from literature sources. Calculation methods for the hydrodynamic radius vary and include size exclusion chromatography, intrinsic viscosity measurement, molecular dynamic simulations, and light scattering.

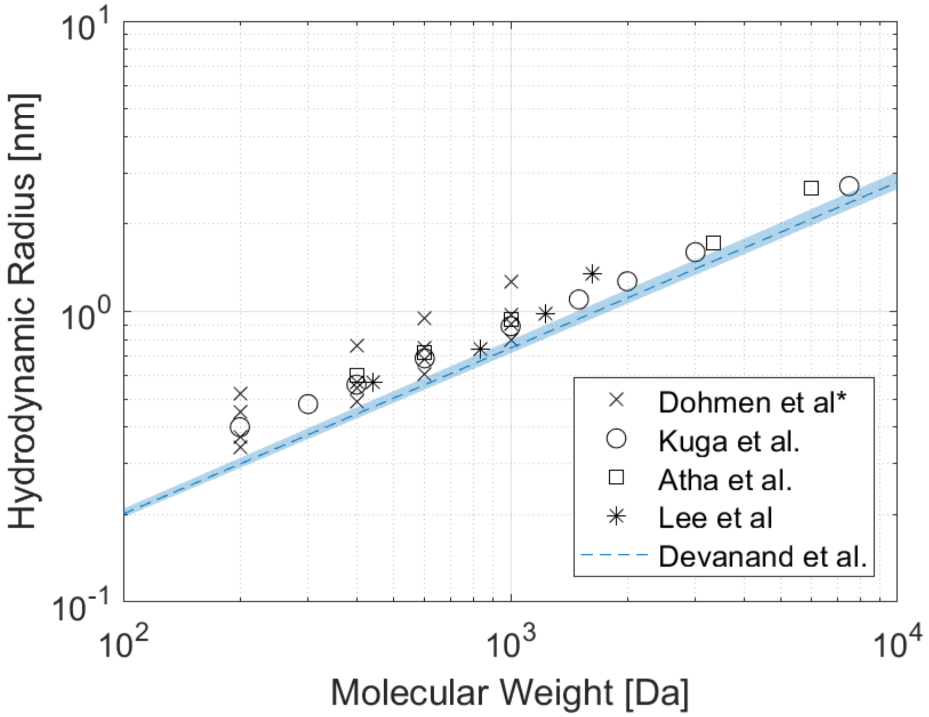

Figure S8: Hydrodynamic radius of PEG in dilute regime. The values are from literature. The dashed line and its boundary are based on a relationship between the radius and molecular weight from Devanand et al.[22] A boundary shows the errors of their fittings. ($R_h[nm] = 0.0145MW^{0.571\pm0.009}$).[22] Dohnen et al.[29]determined the hydrodynamic radius based on the intrinsic viscosity versus molecular weight relation. They also referred to the data of others. Kuga et al.[30] ($\circ$) determined hydrodynamic radius GPC connected with a viscometer. Atha et al.[31]($\Box$) and Lee et al.[32]($*$) show the results of the computational simulations.

S3.4 Critical Concentration

After a specific concentration, the polymer units begin to overlap. This threshold is known as the critical concentration. The critical concentration is determined by using eq. 13, which assumes that each polymer takes up an average volume of ($V = 4/3\pi R_g^3$). The equation employs M for molecular weight, N_A for Avagadro's number, and R_g for the polymer's radius of gyration. This concentration has a unit of g/m^3, and it is converted to mol/m^3 by dividing the molecular weight of the polymer M. The results are given in Figure S9. The below region of the black line indicates the dilute regime, while the area above the black line shows the semi-dilute regime. The red crosses indicate the concentration used in this study.

$$c^* = \frac{M}{N_A \frac{4}{3}\pi R_g^3}, \tag{13}$$

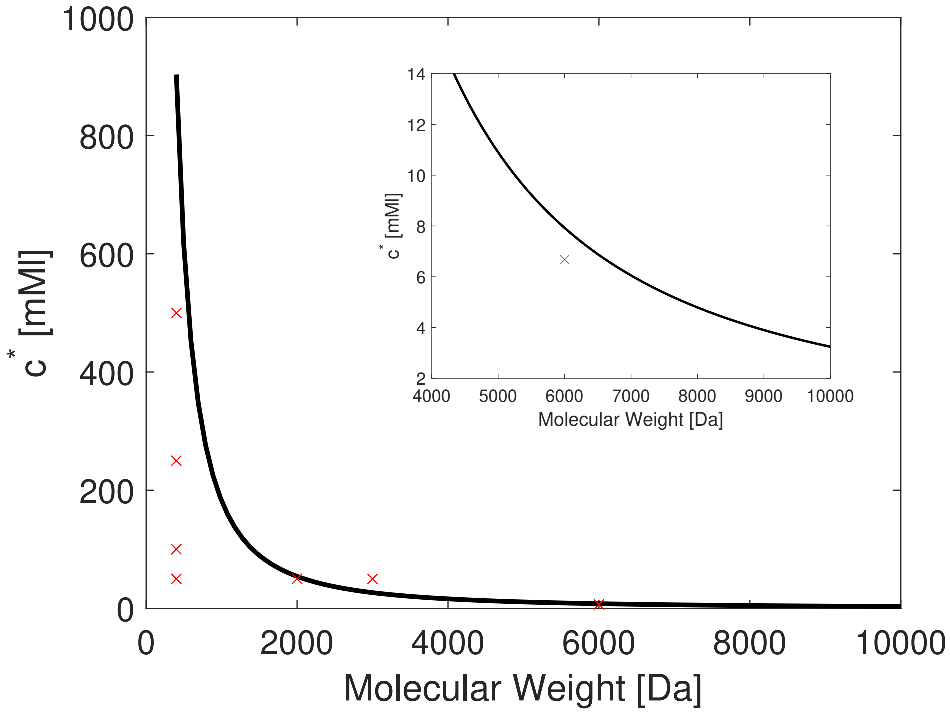

Figure S9: Critical molar concentration of PEG over molecular weights. The values were determined by using eq 13. The below black line shows the dilute regime, and above the black line indicates the semi-dilute regime. The red crosses show the concentration values used in the study.

S4 Dialysis of the Polyelectrolyte Solutions

We performed dialysis on the polyelectrolyte solution to remove any trace amounts of ions from the solution. We used two different dialysis membranes, one with a molecular weight cut-off 14,000 Da for NaPSS 70,000 MW, and a molecular weight cut of 100,000 Da for NaPSS 1,000,000 Da. We performed the diffusiophoresis experiments with dialysis and without dialysis.

S4.1 Diffusiophoresis Results of the Polyelectrolytes without Dialysis

The diffusiophoretic analysis was repeated without using a purification step. The results can be seen as images in Figure S10 and as intensity analysis in Figure S11. For the analysis, we have used three different concentrations and observed three different phases (evacuation, stationary, and exclusion phases).

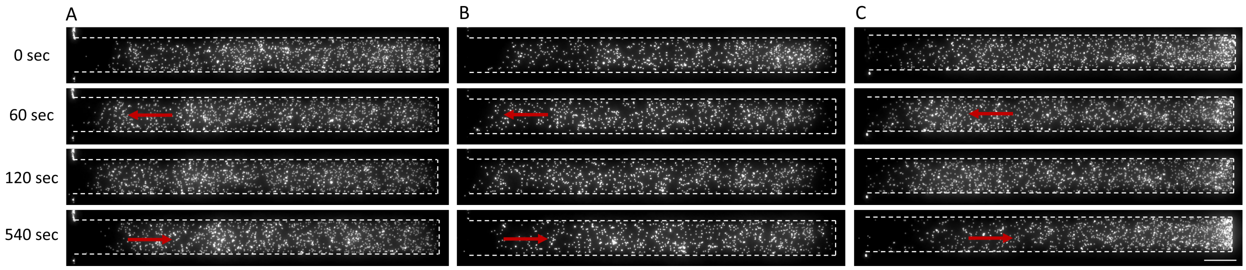

Figure S10: The diffusiophoretic results of the polyelectrolyte solutions (NaPSS - 70,000 Da) without purification phase. The microscope images were taken after contact with polyelectrolyte solutions and the particle solutions at 60 secs, 120 secs, and 540 secs. The concentration of the polyelectrolyte is changed (A) 1 µM (0.07 g/L), (B) 5 µM (0.35 g/L), and (C) 10 µM (0.7 g/L).

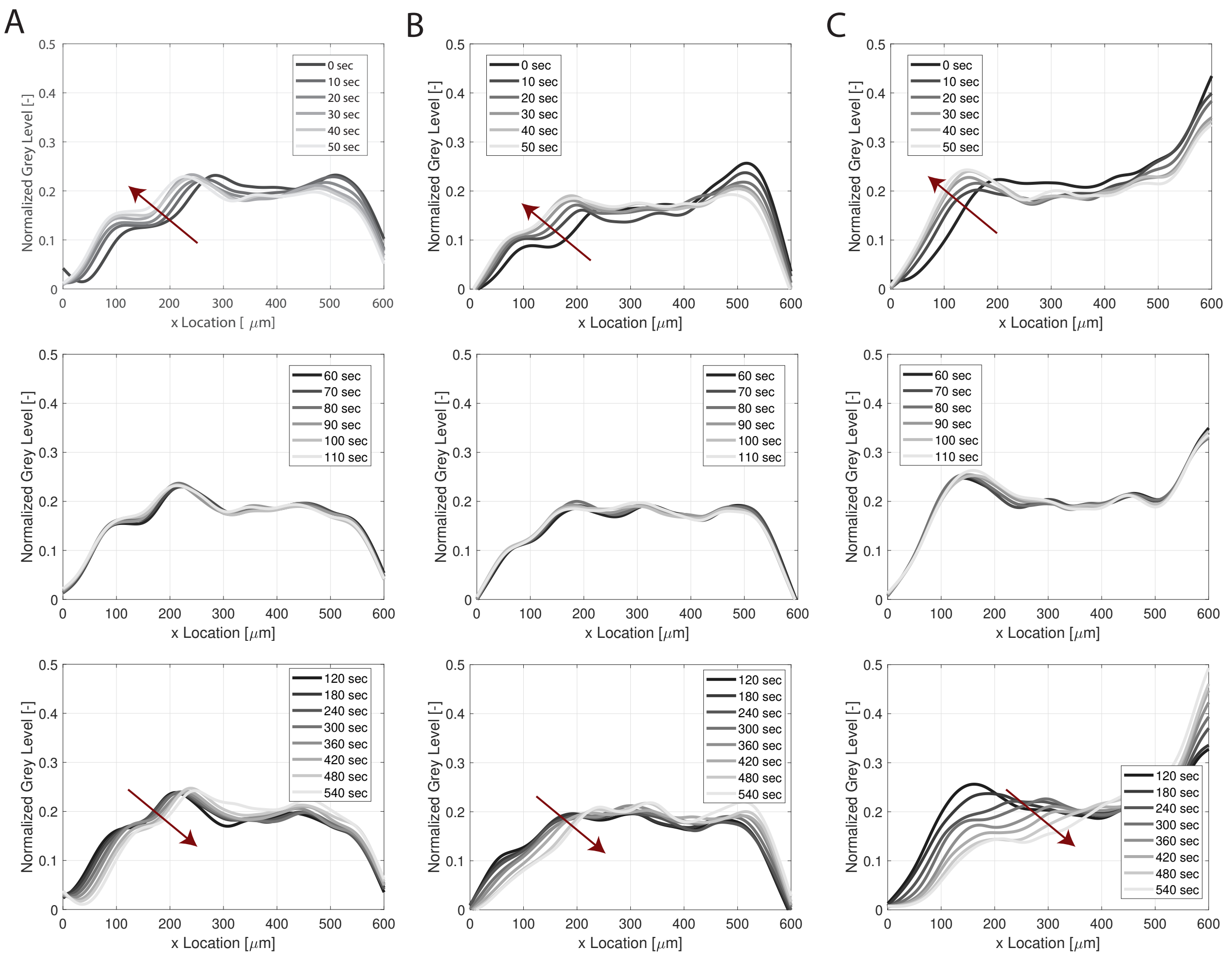

Figure S11: The analysis of the diffusiophoretic experiments (0-540 seconds) performed without the purification step of the NaPSS solutions. The experimental microscope images are shown in Figure S10. The concentration of the NaPSS (70,000 Da) is (A) 1 µM (0.07 g/L), (B) 5 µM (0.35 g/L), and (C) 10 µM (0.7 g/L). The first row (evacuation phase) of the figure represents the normalized grey value of 0-50 seconds with 10 second intervals. The second row shows the normalized grey value at 60 - 110 seconds with 10 second intervals (stationary phase). The last row shows the 120 seconds - 540 seconds of the experiment with a 60 second intervals (exclusion phase).

S4.2 Ion Chromatography Results of Permeate of Dialysis

NaPSS is purified with cleaning steps by using dialysis bags. We analyzed the ions on the permeate side of the dialysis bag to determine the type and the amount of trace ions by using the ion chromatography setup discussed in the experimental section. The results are shown in S12.

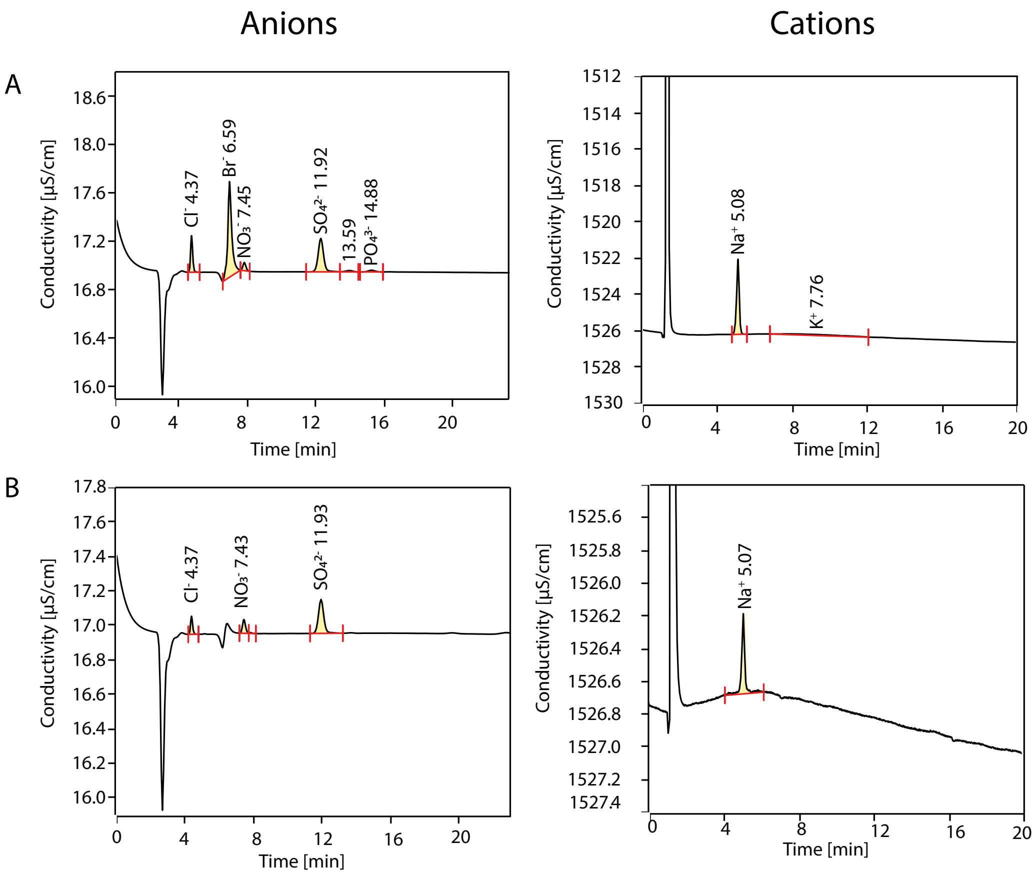

Figure S12: Polyelectrolyte solutions were purified by the dialysis method. The ion chromatography results show the types of ions found in the permeate of the dialysis bag. The analysis of (A) 70,000 Da NaPSS and (B) 1,000,000 Da NaPSS solution is shown separately for anions and cations.

S5 Labelled NaPSS Analysis

The diffusion experiment was performed for 1 g/L of labelled NaPSS 70,000 Da. In the experimental setup, the dead-end channel was filled with only MilliQ water, and the main channel was filled with NaPSS solution. The images were recorded at 1 fps for 60 minutes. The specific part of the channel (after x ≈ 35 μm) was recorded since the main channel was oversaturated and affected the recording setting.

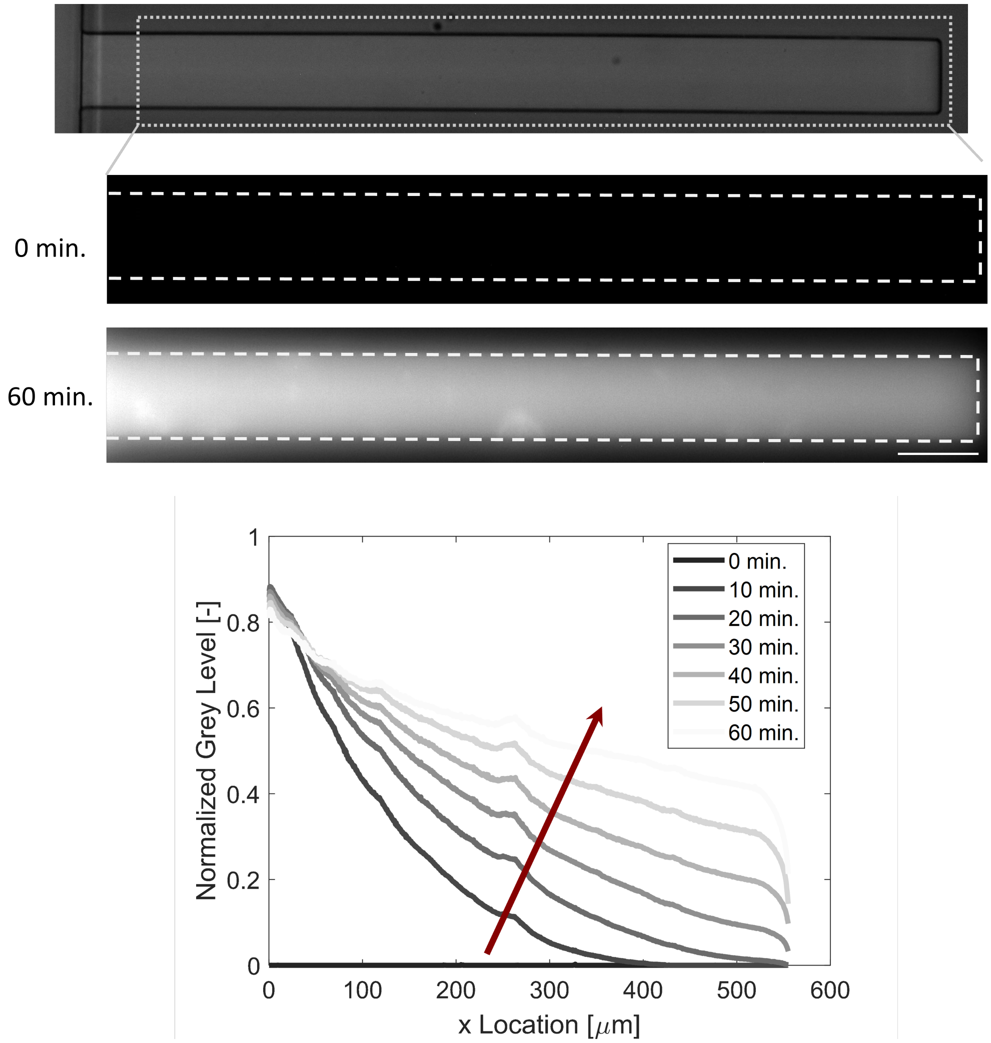

Figure S13: The diffusion experiment was performed with 1 g/L of labelled NaPSS 70,000 Da. The bright field is shown in the upper part of the figure. Below are the microscope images of the experiment at 0 minutes and 60 minutes. Scale bar = 50 μm. At the bottom of the figure, normalized grey value versus x location is given for 10 minute intervals.

S6 Analysis of 1 g/L of 1,000,000 Da PEG

The diffusiophoresis experiment was repeated for the case of 1 g/L of PEG 1,000,000. In the experimental setup, the dead-end channel was filled with 1 µm PS particles in MilliQ water, and the main channel was filled with PEG solution. The images were recorded for 60 minutes at 1 fps.

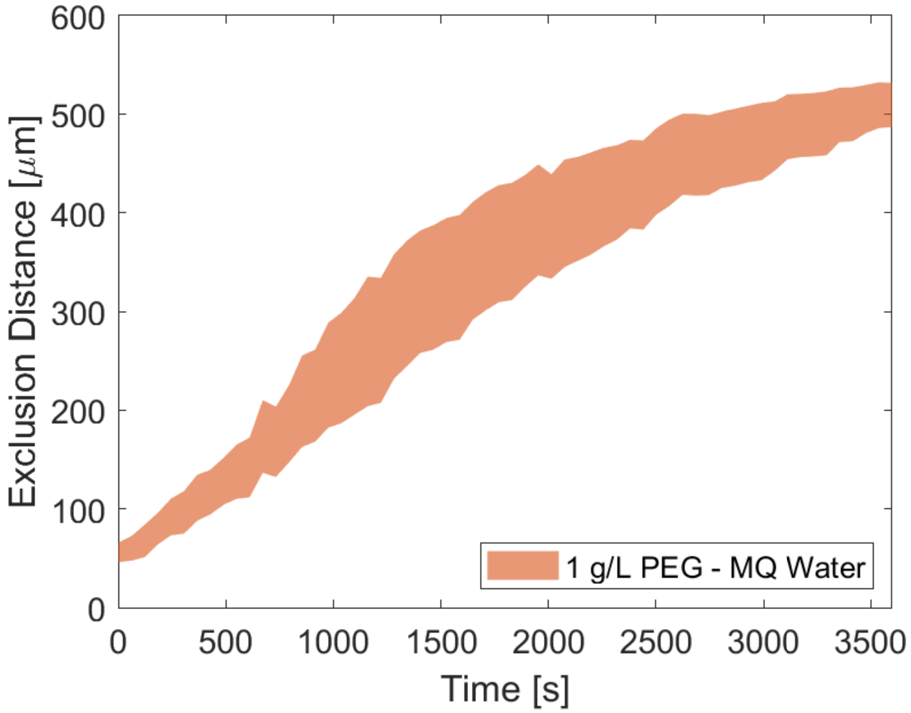

Figure S14: The results of diffusiophoretic experiments performed with 1 g/L of 1,000,000 Da PEG solution. The shaded area indicates the standard error of three experiments.

S7 Simulations with PEG Gradient

Here, we show important aspects of the simulations, such as normalized concentration, absolute gradient, and viscophoretic velocity in a dead-end channel.

S7.1 PEG Concentration Profile Inside Dead-end Channel

The concentration of PEG 400 Da changes over time and space in a dead-end channel. Figure S15 illustrates the concentration profile of PEG 400 Da across x Location in the dead-end channel system at various time intervals. The simulation results of 50 mM, 100 mM, 250 mM, and 500 mM of PEG 400 Da are presented, indicating the changes in concentration.

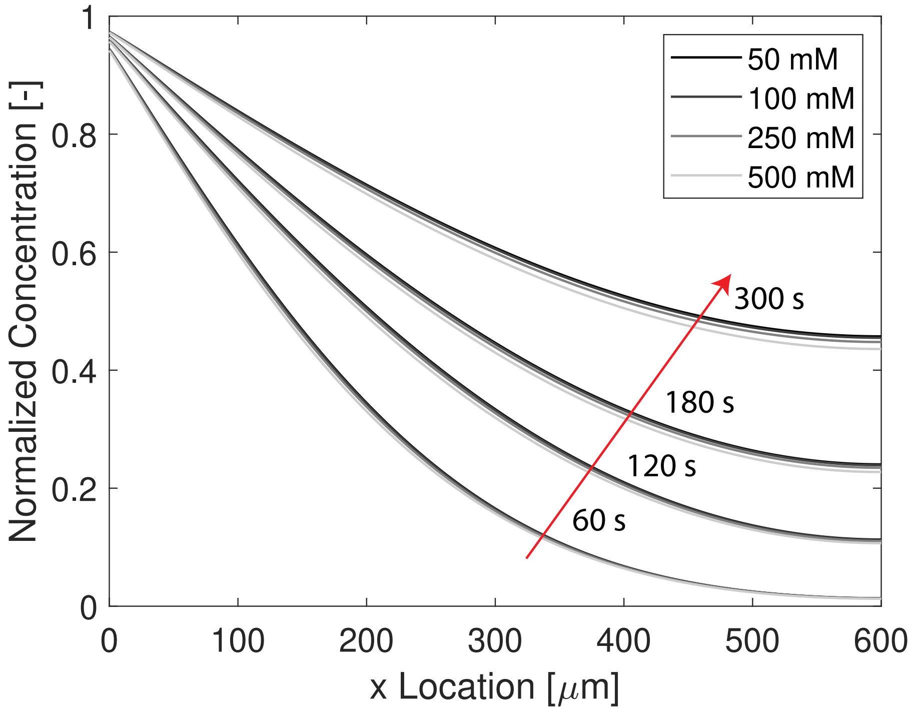

Figure S15: The normalized concentration of PEG 400 Da in a dead-end channel based on the performed simulations. 50 mM, 100 mM, 250 mM, and 500 mM of PEG 400 Da results were given in 60 s, 120 s, 180 s, and 300 s.

S7.2 PEG Absolute Gradient Inside Dead-end Channel

The driving force of non-electrolyte diffusiophoretic velocity is the absolute gradient. Here, we show the absolute gradient of the system. Figure S16 shows the absolute gradient in the dead-end channel system at 60 s and 300 s for concentrations of 50 mM, 100 mM, 250 mM, and 500 mM. Moreover, Figure S17 indicates the absolute gradient with respect to exclusion distance results that we showed in the main text. The gradient of PEG within a dead-end channel is not constant; instead, there is a marked change in values that scales with the concentration.

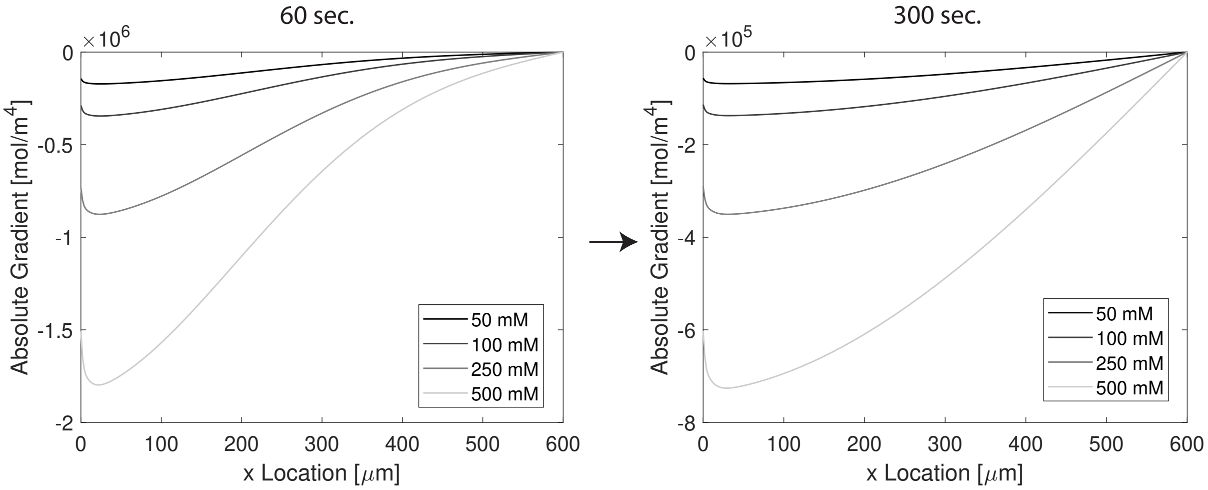

Figure S16: Absolute gradient inside a dead-end channel over x location and time (60 s and 300 s) for concentrations of 50 mM, 100 mM, 250 mM, and 500 mM.

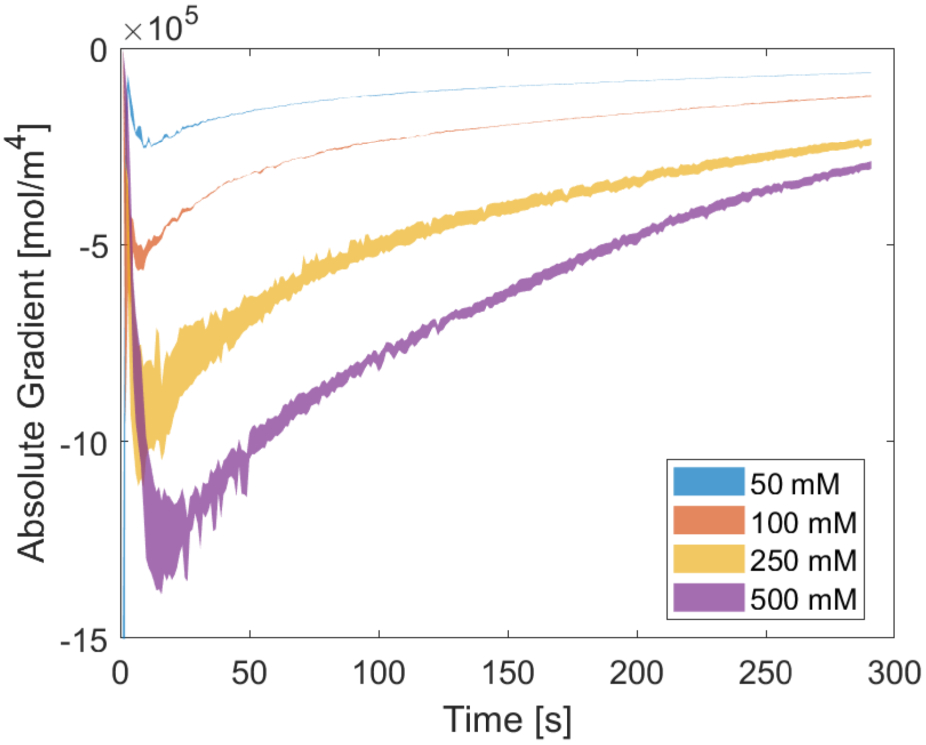

Figure S17: Absolute gradient based on exclusion distance analysis for concentrations of 50 mM, 100 mM, 250 mM, and 500 mM over time. The shaded area shows the standard error of the determination.

S7.3 Viscophoretic Velocity

Based on exclusion distance analysis, we determined the viscophoretic velocity[33] by considering the viscosity change in the system. The viscophoretic velocity was determined considering the diffusivity change of the particle due to viscosity change.

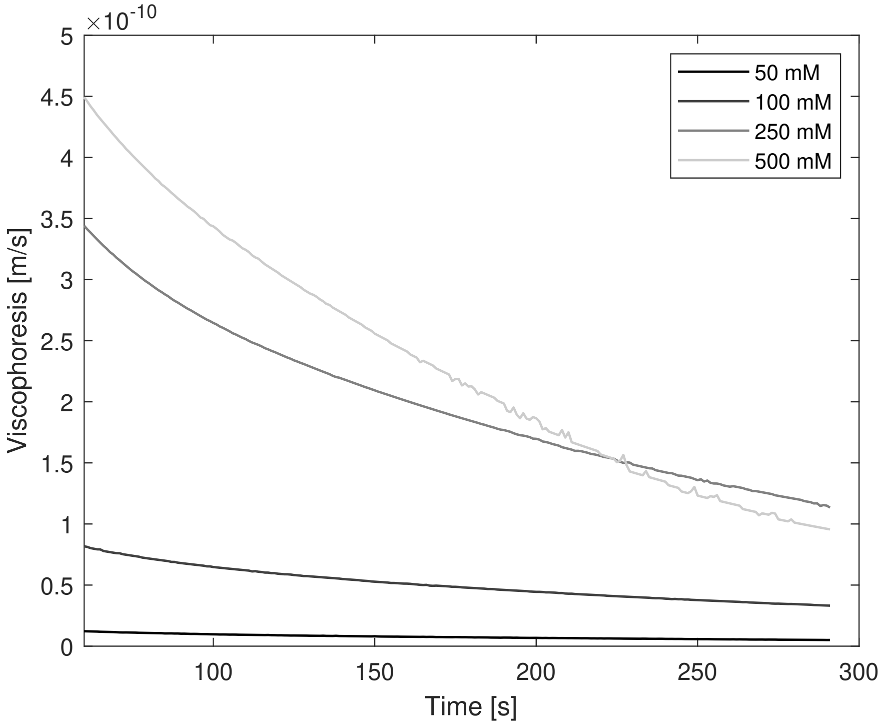

Figure S18: Viscophoretic velocity based on exclusion distance analysis of an experiment.

S7.4 Sensitivity Analysis of Simulations

The polymer radius highly influences the diffusiophoretic velocity for non-electrolytes ($\sim R^2$). Experiments were conducted in a dead-end channel using MQ water with particles and in the main channel with a PEG 6000 Da concentration of 6.67 mM - 40 g/L. We performed three simulations by considering the polymer radius as 4.4 nm, 5.0 nm, and 5.4 nm. The exclusion distance results are given in Figure S19.

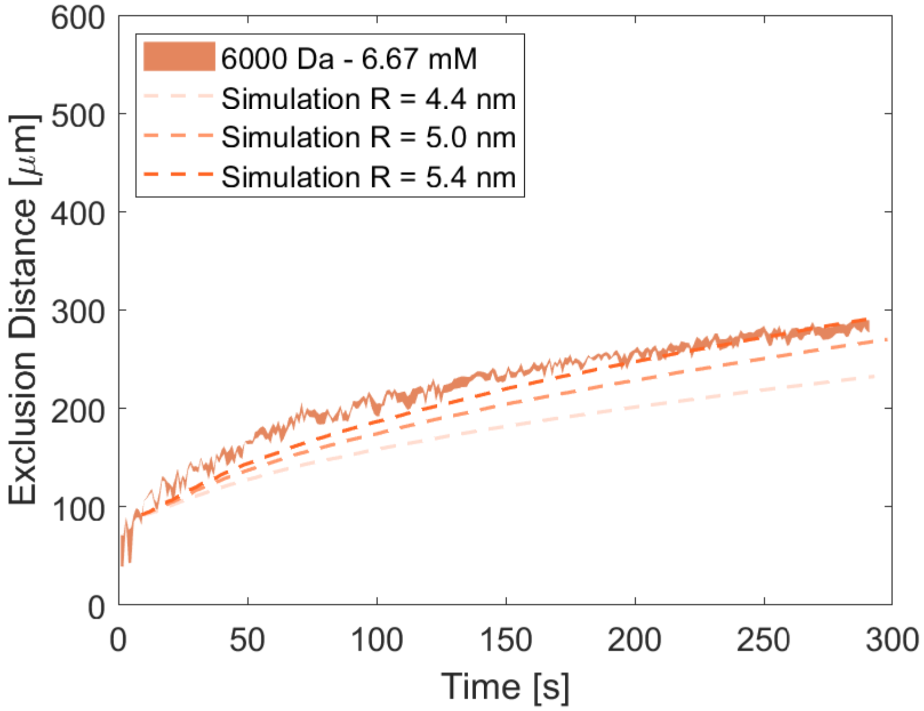

Figure S19: Sensitivity analysis of simulations. Simulations depend on the polymer radius parameter. Three simulations were performed with varied polymer radius values.

S8 Experiments with the Same Mass Concentration of PEG

PEG molecular weight experiments were conducted using the same molar concentrations (50 mM) of different PEG molecular weights. The mass concentration significantly differed between them. For a better understanding of the effect at the same mass concentration, additional experiments were carried out using 40 g/L mass concentrations of PEG with molecular weights of 400 Da and 6000 Da. The exclusion distance analysis is presented in Figure S20.

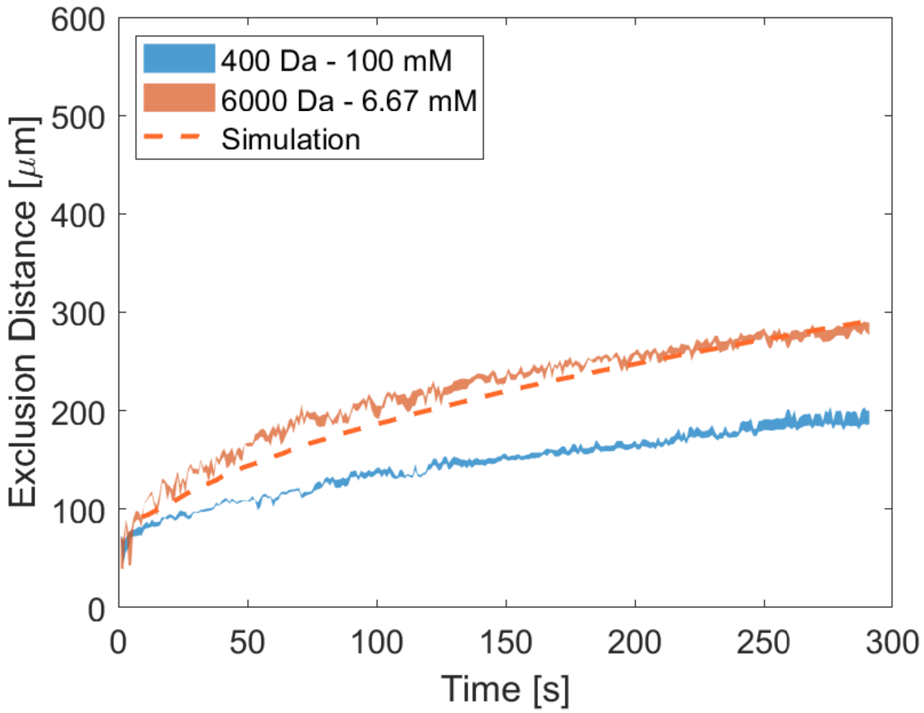

Figure S20: Diffusiophoretic experimental results of using same weight concentration in different molecular weights of PEG. We used 40 g/L of PEG 400 Da and 6000 Da (the molar concentrations are given in the legend). The shaded areas show the standard errors of the three experimental results.

S9 Experiments with Background Salt - PEG

We conducted an additional experiment under a background salt concentration similar to the polyelectrolyte case. As a background salt, 10 mM NaCl is added. The dead-end channel contains PS particles in 10 mM NaCl, while the main channel contains 50 mM of (150 g/L) PEG 3000 in 10 mM NaCl. The exclusion distance analysis with and without added salt is given in Figure S21.

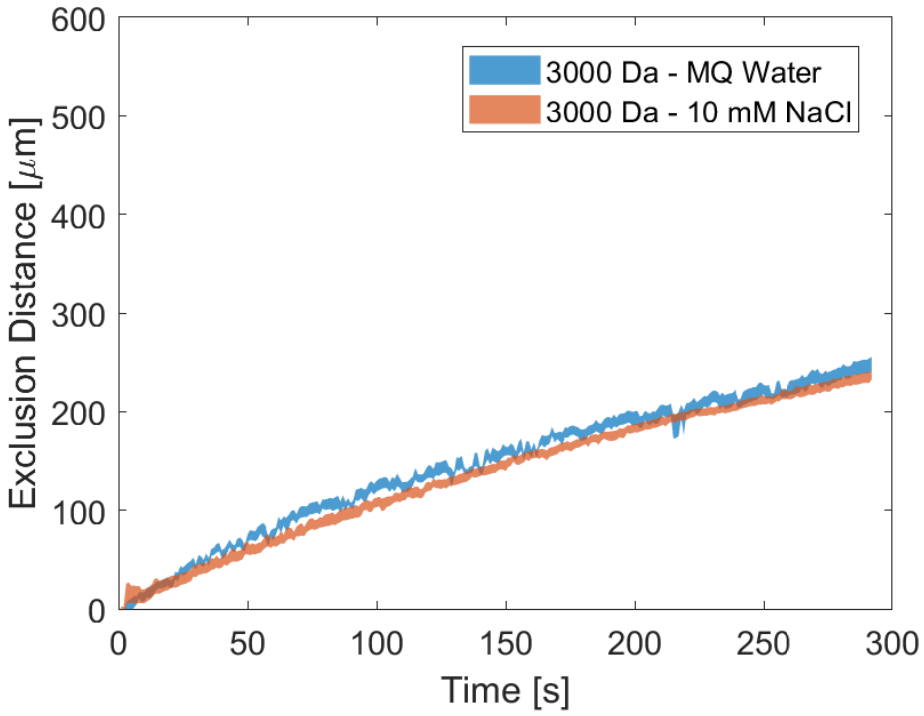

Figure S21: Diffusiophoretic experimental results of using background salt. The initial starting point was normalized. The shaded areas show the standard errors of the three experimental results.

S10 SEM Images of Silica Particles

SEM images of the silica nanoparticles are shown in Figure S22. The radius determined from the SEM images is 10.3 ± 1.0 (over 100 particles), which is close to that measured by the dynamic light scattering (DLS) method.

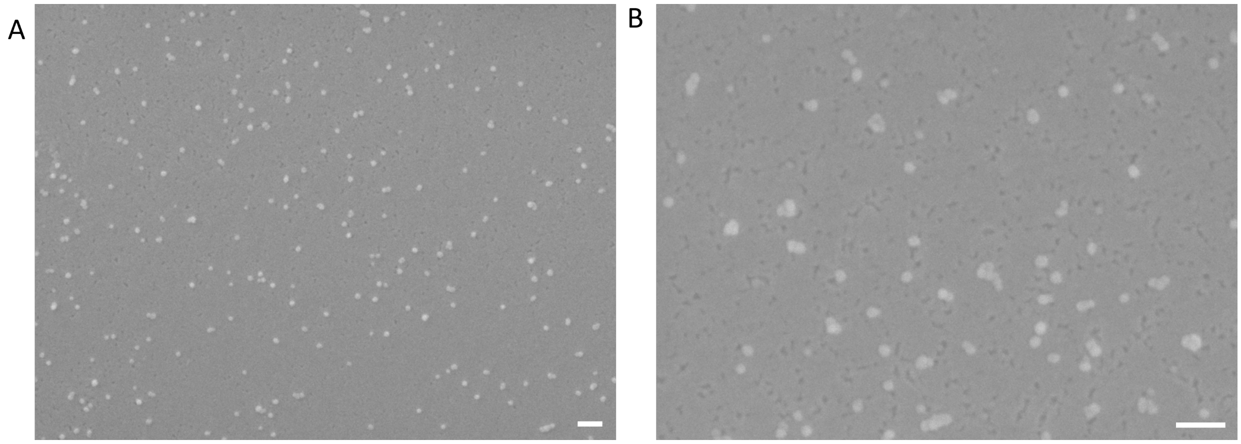

Figure S22: The SEM images of the silica nanoparticles. The magnification was set to (A) 50,000x and (B) 100,000x. Scale bar = 100 nm.

S11 Simulation Results of Silica Nanoparticle Gradient for Different Gradients

The experiments with silica nanoparticle gradients were attempted to explain by the ZJD model.[27] The procedure is given in the above section S1.4. The simulation was performed for the different volume fractions of the particles (ϕ_1), and the results are shown in Figure S23.

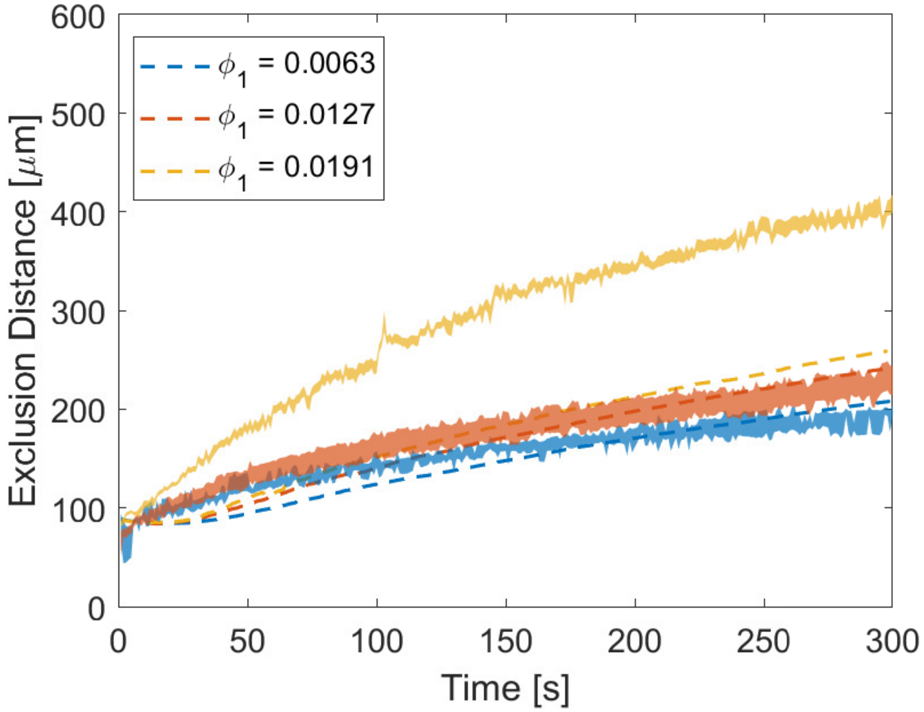

Figure S23: The results of the ZJD model for three different volume fractions (ϕ_1) of the silica particles. The simulation results are shown as dashed lines. The color of the dashed lines and the experimental results represent the same volume fractions. The shaded area shows the experimental results with the standard error of at least three experiments.

S12 Simulation Results of Silica Nanoparticle Gradients with Varied PS Fractions

The cross-interaction simulation is tested with different volume fractions of PS particles. Figure S24 shows that the PS volume fraction does not influence the exclusion distance.

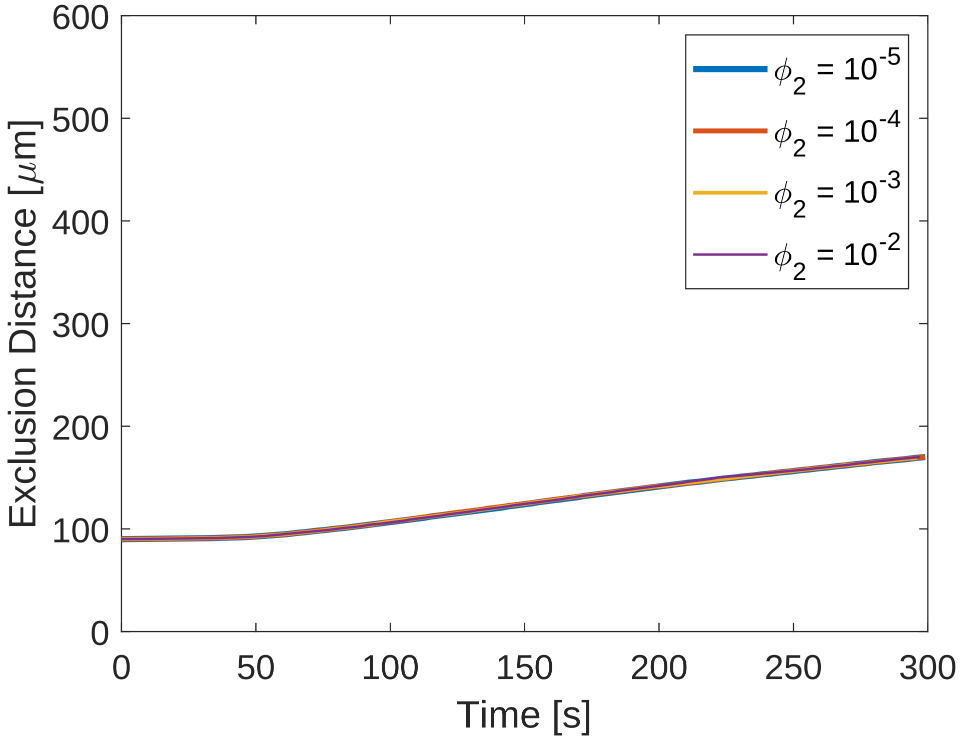

Figure S24: The simulation results of the exclusion distance versus time. The initial volume fraction of the PS particle is changed while while keeping the other parameters.

S13 Simulation and Experimental Results of Smaller Silica Nanoparticle Gradients

We repeated the diffusiophoretic experiments using silica particles with a radius of 15 nm. The microscope images 300 seconds after the contact is given in Figure S25.

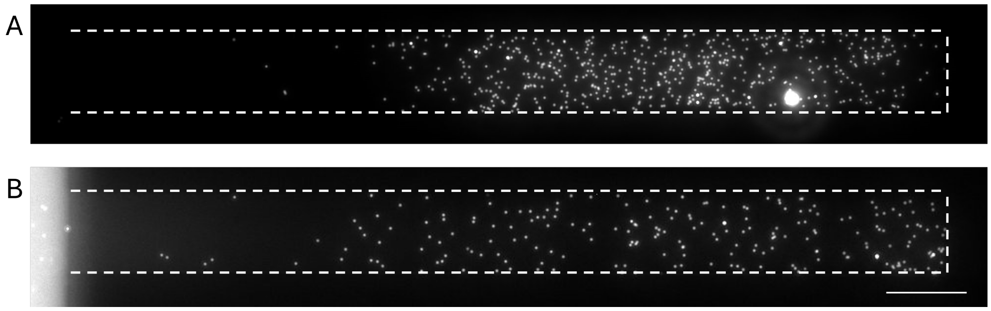

Figure S25: Diffusiophoretic experiments with different sizes of the silica particles at constant initial volume fractions (= 0.013). The radius of the silica particles is approximately (A) 9 nm, and (B) 15 nm (fluorescent). The microscope images were taken after 300 seconds of contact. Scale bar = 50 µm.

The cross-interaction theory for binary colloidal mixtures is repeated for different values of $\alpha = R_{PS}/R_{silica}$. The exclusion distance increases as the ratio between the particle sizes increases. Figure S26 shows exclusion distance with different α values.

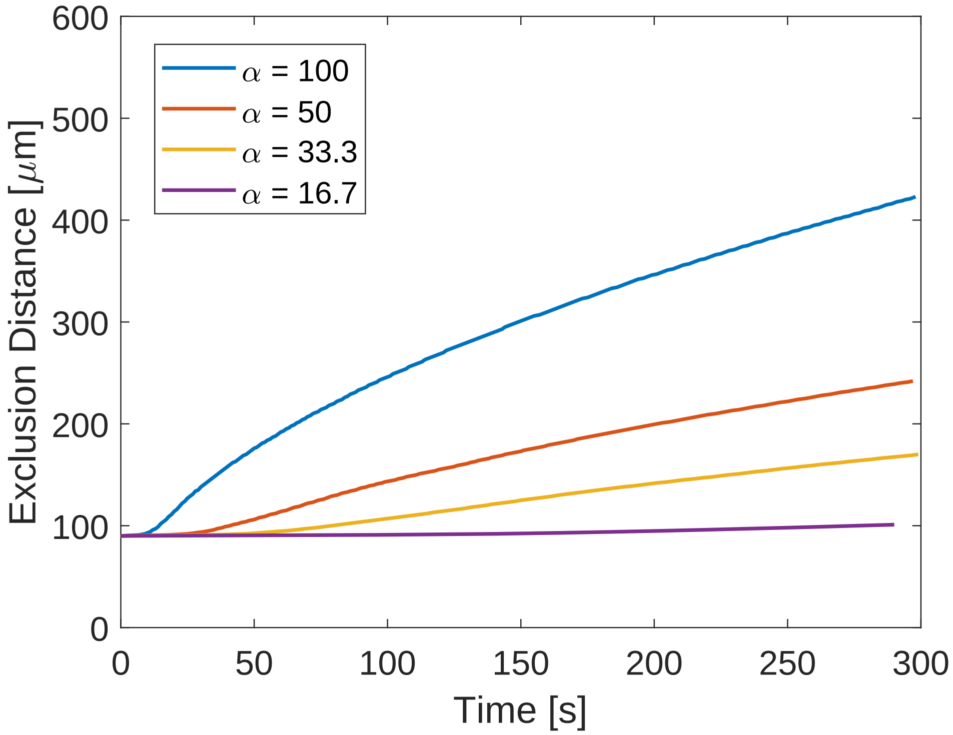

Figure S26: Exclusion distance versus time for different α values. To change the α values, R1 is changed to 5 nm ($\alpha = 100$), 10 nm ($\alpha = 50$), 15 nm ($\alpha = 33.3$), and 30 nm ($\alpha = 16.7$).

References

(1) Anderson, J. L.; Lowell, M. E.; Prieve, D. C. Motion of a particle generated by chemical gradients Part 1. Non-electrolytes. *J. Fluid Mech.* **1982**, *117*, 107–121.

(2) Velegol, D.; Garg, A.; Guha, R.; Kar, A.; Kumar, M. Origins of concentration gradients for diffusiophoresis. *Soft Matter* **2016**, *12*, 4686–4703.

(3) Sear, R. P.; Warren, P. B. Diffusiophoresis in nonadsorbing polymer solutions: The Asakura-Oosawa model and stratification in drying films. *Phys. Rev. E* **2017**, *96*, 062602.

(4) Anderson, J. Colloid Transport By Interfacial Forces. *Annu. Rev. Fluid Mech.* **1989**, *21*, 61–99.

(5) Marbach, S.; Yoshida, H.; Bocquet, L. Local and global force balance for diffusiophoretic transport. *J. Fluid Mech.* **2020**, *892*, A6.

(6) Collins, M.; Mohajerani, F.; Ghosh, S.; Guha, R.; Lee, T.-H.; Butler, P. J.; Sen, A.; Velegol, D. Nonuniform Crowding Enhances Transport. *ACS Nano* **2019**, *13*, 8946–8956.

(7) Ninni, L.; Burd, H.; Fung, W. H.; Meirelles, A. J. A. Kinematic Viscosities of Poly(ethylene glycol) Aqueous Solutions. *J. Chem. Eng. Data* **2003**, *48*, 324–329.

(8) Kirinčič, S.; Klofutar, C. A volumetric study of aqueous solutions of poly(ethylene glycol)s at 298.15 K. *Fluid Phase Equilibria* **1998**, *149*, 233–247.

(9) Peppin, S. Effective hard-sphere model of diffusion in aqueous polymer solutions. *viXra preprint:viXra2102.0065* **2021**, vixra.org/abs/2102.0065.

(10) Vergara, A.; Paduano, L.; Sartorio, R. Multicomponent Diffusion in Systems Containing Molecules of Different Size. 4. Mutual Diffusion in the Ternary System Tetra(ethylene glycol) Di(ethylene glycol) Water. *J. Phys. Chem. B* **2001**, *105*, 328–334.

(11) Kar, A.; Chiang, T.-Y.; Ortiz Rivera, I.; Sen, A.; Velegol, D. Enhanced Transport into and out of Dead-End Pores. *ACS Nano* **2015**, *9*, 746–753.

(12) Shim, S.; Nunes, J. K.; Chen, G.; Stone, H. A. Diffusiophoresis in the presence of a pH gradient. *Phys. Rev. Fluids* **2022**, *7*, 110513.

(13) Akdeniz, B.; Wood, J. A.; Lammertink, R. G. Diffusiophoresis and Diffusio-osmosis into a Dead-End Channel: Role of the Concentration-Dependence of Zeta Potential. *Langmuir* **2023**, *39*, 2322–2332.

(14) Ault, J. T.; Warren, P. B.; Shin, S.; Stone, H. A. Diffusiophoresis in one-dimensional solute gradients. *Soft Matter* **2017**, *13*, 9015–9023.

(15) Ault, J. T.; Shin, S.; Stone, H. A. Diffusiophoresis in narrow channel flows. *J. Fluid Mech.* **2018**, *854*, 420–448.

(16) Shin, S.; Ault, J. T.; Feng, J.; Warren, P. B.; Stone, H. A. Low-Cost Zeta Potentiometry Using Solute Gradients. *Adv. Mater.* **2017**, *29*, 1701516.

(17) Shin, S. Diffusiophoretic separation of colloids in microfluidic flows. *Phys. Fluids* **2020**, *32*, 101302.

(18) Gupta, A.; Shim, S.; Stone, H. A. Diffusiophoresis: from dilute to concentrated electrolytes. *Soft Matter* **2020**, *16*, 6975–6984.

(19) Battat, S.; Ault, J. T.; Shin, S.; Khodaparast, S.; Stone, H. A. Particle entrainment in dead-end pores by diffusiophoresis. *Soft Matter* **2019**, *15*, 3879–3885.

(20) Wilson, J. L.; Shim, S.; Yu, Y. E.; Gupta, A.; Stone, H. A. Diffusiophoresis in Multivalent Electrolytes. *Langmuir* **2020**, *36*, 7014–7020.

(21) Vergara, A.; Paduano, L.; Vitagliano, V.; Sartorio, R. Mutual diffusion in aqueous solution of poly(ethyleneglycol) samples. Some comments on the effect of chain length and polydispersity. *Phys. Chem. Chem. Phys.* **1999**, *1*, 5377–5383.

(22) Devanand, K.; Selser, J. C. Asymptotic behavior and long-range interactions in aqueous solutions of poly(ethylene oxide). *Macromolecules* **1991**, *24*, 5943–5947.

(23) Gupta, A.; Rallabandi, B.; Stone, H. A. Diffusiophoretic and diffusioosmotic velocities for mixtures of valence-asymmetric electrolytes. *Phys. Rev. Fluids* **2019**, *4*, 043702.

(24) Sear, R. P. Stratification of mixtures in evaporating liquid films occurs only for a range of volume fractions of the smaller component. *J. Chem. Phys.* **2018**, *148*.

(25) Staffeld, P. O.; Quinn, J. A. Diffusion-induced banding of colloid particles via diffusiophoresis: 1. Electrolytes. *J. Colloid Interface Sci.* **1989**, *130*, 69–87.

(26) Rees-Zimmerman, C. R.; Chan, D. H.; Armes, S. P.; Routh, A. F. Diffusiophoresis of latex driven by anionic nanoparticles and their counterions. *J. Colloid Interface Sci.* **2023**, *649*, 364–371.

(27) Zhou, J.; Jiang, Y.; Doi, M. Cross Interaction Drives Stratification in Drying Film of Binary Colloidal Mixtures. *Phys. Rev. Lett.* **2017**, *118*, 108002.

(28) Albright, J. G.; Paduano, L.; Sartorio, R.; Vergara, A.; Vitagliano, V. Multicomponent Diffusion in Systems Containing Molecules of Different Size. 1. Mutual Diffusion in the Ternary System Poly(ethylene glycol) 2000 + Poly(ethylene glycol) 200 + Water. *J. Chem. Eng. Data* **2001**, *46*, 1283–1291.

(29) Dohmen, M. P. J.; Pereira, A. M.; Timmer, J. M. K.; Benes, N. E.; Keurentjes, J. T. F. Hydrodynamic Radii of Polyethylene Glycols in Different Solvents Determined from Viscosity Measurements. *J. Chem. Eng. Data* **2008**, *53*, 63–65.

(30) Kuga, S. Pore size distribution analysis of gel substances by size exclusion chromatography. *J. Chromatogr. A* **1981**, *206*, 449–461.

(31) Atha, D.; Ingham, K. Mechanism of precipitation of proteins by polyethylene glycols. Analysis in terms of excluded volume. *J. Biol. Chem.* **1981**, *256*, 12108–12117.

(32) Lee, H.; Venable, R. M.; MacKerell, A. D.; Pastor, R. W. Molecular Dynamics Studies of Polyethylene Oxide and Polyethylene Glycol: Hydrodynamic Radius and Shape Anisotropy. *Biophys. J.* **2008**, *95*, 1590–1599.

(33) Khandan, V.; Boerkamp, V.; Jabermoradi, A.; Fontana, M.; Hohlbein, J.; Verpoorte, E.; Chiechi, R. C.; Mathwig, K. Viscophoretic particle transport. *arXiv preprint arXiv:2212.11503* **2022**, 10.48550/arXiv.2212.11503.
